# Plasma exosomal miR-25-3p alleviates acute pancreatitis-associated lung injury by inhibiting ferroptosis of type II alveolar epithelial cells *via* NOX4/NCOA4-mediated ferritinophagy

**DOI:** 10.1515/jtim-2026-0071

**Published:** 2026-10-23

**Authors:** Zixin Wang, Zijian Huang, Jiafu Wang, Liang Zhang, Long Cheng, Meng He, Xuchen Hu, Junpeng Wu, Yusen Feng, Liang Ji, Yan Jin, Gang Wang

**Affiliations:** Department of Oncology Laparoscopic Surgery, The First Affiliated Hospital of Harbin Medical University, Harbin, Heilongjiang Province, China; Key Laboratory of Hepatosplenic Surgery, Ministry of Education, The First Affiliated Hospital of Harbin Medical University, Harbin, Heilongjiang Province, China; Department of Breast Surgery, The First Affiliated Hospital of Harbin Medical University, Harbin, Heilongjiang Province, China; Department of Nuclear Medicine, The First Affiliated Hospital of Harbin Medical University, Harbin, Heilongjiang Province, China; Department of Gastrointestinal Surgery, The First Affiliated Yijishan Hospital of Wannan Medical College, Wuhu, Anhui Province, China

**Keywords:** acute pancreatitis-associated lung injury, plasma exosomal microRNA, NADPH oxidase 4, ferroptosis, ferritinophagy

## Abstract

**Background and Objectives:**

Acute pancreatitis-associated lung injury (AP-LI) is a severe complication with limited therapeutic options. This study investigated the role of plasma exosomes in AP-LI and the underlying molecular mechanism.

**Methods:**

A murine AP-LI model and primary alveolar type II epithelial (AT2) cells were used. Small RNA sequencing, bioinformatic and enrichment analyses, functional assays, and *in vivo* and *in vitro* rescue experiments were performed to identify key exosomal miRNAs and define their downstream signaling pathways.

**Results:**

Plasma exosomes aggravated AP-LI. Small RNA sequencing revealed marked alterations in exosomal miRNA profiles, with miR-25–3p identified as a key candidate associated with oxidative stress and ferroptosis. NADPH oxidase 4 (NOX4) was predicted and validated as a direct target of miR-25–3p. miR-25–3p suppressed NOX4 expression and attenuated ferroptosis in AT2 cells. Nuclear receptor coactivator 4 (NCOA4)-mediated ferritinophagy acted downstream of NOX4, promoted iron dysregulation, and reinforced ferroptotic injury. Rescue experiments confirmed the protective role of the miR-25–3p/NOX4/NCOA4 axis.

**Conclusions:**

Plasma exosomal miR-25–3p alleviates AP-LI by repressing NOX4 and limiting NCOA4-mediated ferritinophagy and ferroptosis, suggesting a potential therapeutic target for pulmonary complications of acute pancreatitis.

## Introduction

Acute pancreatitis (AP) is a rapidly progressive inflammatory disorder characterized by pancreatic autodigestion that can progress to severe systemic inflammatory response syndrome (SIRS) and multiple organ dysfunction syndrome (MODS).^[1]^ Among patients with severe AP, 15%-60% develop acute pancreatitis-associated lung injury (AP-LI), a life-threatening complication that frequently culminates in respiratory failure. Despite its clinical importance, the molecular basis of AP-LI remains incompletely defined.^[2,3]^

Ferroptosis is a regulated form of cell death that is distinct from apoptosis and necrosis and is characterized by iron dependence, lipid peroxidation, and characteristic mitochondrial abnormalities.^[4]^ Aberrant ferroptosis has been widely documented in experimental AP.^[5,6]^ During AP, acinar cell injury and necrosis trigger the release of digestive enzymes and inflammatory mediators, leading to both local pancreatic damage and systemic inflammation. Oxidative stress, disordered iron homeostasis, and disturbed lipid metabolism are central pathological features of AP and are also key drivers of ferroptosis. Beyond the pancreas, ferroptosis is increasingly recognized as a contributor to secondary organ injury, including injury to the liver, kidney, and lung. By amplifying the release of proinflammatory cytokines, damage-associated molecular patterns (DAMPs), and iron-related mediators, ferroptosis may promote the development of SIRS and MODS.

NADPH oxidase 4 (NOX4), a member of the NADPH oxidase family, is an important source of reactive oxygen species (ROS) in the lung and has been implicated in acute respiratory distress syndrome (ARDS) and chronic obstructive pulmonary disease (COPD).^[7,8]^ Mechanistically, NOX4 can promote ferroptosis by increasing ROS generation and facilitating intracellular iron accumulation. Ferritinophagy, a selective form of autophagy mediated by Nuclear Receptor Coactivator 4 (NCOA4), degrades ferritin and releases labile Fe^2+^, thereby fueling lipid peroxidation and ferroptotic injury. In this process, the ferritin-NCOA4-Microtubule-associated protein 1 light chain 3 (LC3) complex supports autophagosome-lysosome fusion, ferritin degradation, and ferroptosis execution.^[9, 10, 11]^ In multiple lung injury models, including ventilator-induced and chemically induced injury, activation of NCOA4-dependent ferritinophagy exacerbates pulmonary ferroptosis.^[12]^ Within the pancreatic-lung inflammatory axis, AT2 cells are of particular interest because they are essential for surfactant production and alveolar repair. Clinical and experimental studies indicate that injury or loss of AT2 cells disrupts alveolar-capillary barrier function and impairs gas exchange.^[13]^ AT2-cell ferroptosis has been reported in several models of ischemia-reperfusion and infectious lung injury and directly contributes to structural and functional deterioration.^[14]^

Extracellular vesicles, particularly exosomes, have emerged as key mediators of interorgan communication because of the stability of their membrane structure and the specificity of their molecular cargo, including miRNAs. Exosomal miRNAs have been increasingly implicated in the regulation of ferroptosis in non-neoplastic diseases such as cerebral ischemia, hepatic injury, and renal damage.^[15]^ In AP, dysregulated exosomal miRNAs contribute to disease progression and multiorgan injury by modulating acinar cell death and premature trypsinogen activation.^[16,17]^ Among these miRNAs, miR-25-3p, a member of the miR-106b/25 family, is of particular interest because of its antioxidative, antiapoptotic, and anti-inflammatory properties. miR-25-3p directly targets NOX4, a major intracellular source of ROS, and can modulate ferroptotic sensitivity through the c-Myc/SLC7A11 axis.^[18,19]^ Tumor-derived exosomal miR-25-3p has also been shown to alter vascular permeability and inflammatory signaling through the PI3K/AKT pathway.^[20]^ However, the role of plasma exosomal miR-25-3p in AP-LI has not been defined. Based on these observations, we hypothesized that miR-25-3p acts upstream of NOX4, whereas NCOA4-mediated ferritinophagy acts downstream of NOX4 to promote ferroptosis during AP-LI.

To address this question, we established a murine AP-LI model and combined exosome-tracking experiments with *in vitro* studies in primary AT2 cells. We aimed to determine whether plasma exosomal miR-25-3p alleviates AP-LI by suppressing AT2-cell ferroptosis through the NOX4/NCOA4 ferritinophagy axis. This study was designed to clarify the molecular sequence linking exosome-mediated pancreas-lung communication to oxidative stress, ferritinophagy, and ferroptotic injury, and to evaluate the therapeutic potential of exosomal miRNA-based intervention.

## Materials and methods

### Ethics statement and human blood sample collection

All human peripheral blood specimens utilized in this study were residual discarded clinical specimens collected from the First Clinical College of Harbin Medical University. All experimental procedures involving human blood samples strictly complied with the principles outlined in the Declaration of Helsinki. This research protocol was reviewed and approved by the Institutional Review Board/ Ethics Committee of the First Affiliated Hospital of Harbin Medical University (Protocol Number: IRB-AF/SC-12/03.0). Written informed consent was obtained from all enrolled participants prior to sample collection.

### Inclusion and exclusion criteria

A total of 45 participants were enrolled and divided into three groups with 15 cases in each group. All case screenings strictly followed the criteria below to ensure the homogeneity of research subjects and the reliability of results.

#### Basic requirements for all research subjects

All participants aged 18-75 years, regardless of gender, were able to cooperate with sample collection and clinical data collation. Pregnant and lactating women, as well as patients with mental illness or cognitive impairment who could not cooperate with the study, were excluded.

#### Inclusion criteria for each group

##### (1) Healthy control group (HC group, *n* = 15)

- Underwent a complete health check-up at the hospital's physical examination center, with all indicators within the normal reference range and no organic diseases;
- No history of acute or chronic inflammation, infection, surgery, or trauma in the past 3 months, and no use of anti-inflammatory or immunomodulatory drugs;
- No history of chronic underlying diseases of the pancreas, hepatobiliary system, or malignant tumors.

##### (2) Mild/Mild severe acute pancreatitis group (MAP/ MSAP group, *n* = 15)

- Conformed to the diagnostic criteria for MAP specified in the *Chinese Guidelines for the Diagnosis and Treatment of Acute Pancreatitis (2021 Edition)*: acute onset, persistent upper abdominal pain, serum amylase and/ or lipase levels ≥3 times the upper limit of normal, abdominal imaging (ultrasound/computed tomography [CT]) showing pancreatic edema and inflammation, without pancreatic necrosis, organ failure, or local complications;
- Met the following severity scoring criteria: Acute Physiology and Chronic Health Evaluation II (APACHE II) score <8 points, Ranson score <3 points, and CT Severity Index (CTSI) <4 points;
- No dysfunction of vital organs (respiratory, circulatory, renal systems), no complications such as lung injury, shock, or renal failure, and able to cooperate with sample collection and data collation.

##### (3) Severe acute pancreatitis with lung injury group (AP-LI group, *n* = 15)

- Conformed to the diagnostic criteria for SAP specified in the *Chinese Guidelines for the Diagnosis and Treatment of Acute Pancreatitis (2021 Edition)*: persistent organ failure (duration >48 hours) within 48 hours after the onset of AP, or complicated with local complications such as pancreatic necrosis or abscess. Severity scores met the following criteria: APACHE II score ≥8 points, Ranson score ≥3 points, and CTSI ≥4 points;
- Complicated with acute lung injury (ALI) or acute respiratory distress syndrome (ARDS), in accordance with the *Berlin Definition of Acute Respiratory Distress Syndrome*: onset within 72 hours, bilateral diffuse infiltrates on chest imaging (CT or chest X-ray) excluding cardiogenic pulmonary edema; oxygenation index (PaO_2_/FiO_2_) ≤300 mmHg (ALI) or ≤200 mmHg (ARDS);
- Lung injury was directly induced by AP, excluding lung injury caused by other etiologies such as pulmonary infection, acute exacerbation of chronic obstructive pulmonary disease, and pulmonary embolism.

#### Common exclusion criteria for all groups

1. Failed to meet the inclusion criteria of each group, or incomplete clinical data/sample collection that could not complete the relevant tests of the study.
2. Complicated with special types of pancreatitis (*e.g*., acute exacerbation of chronic pancreatitis, autoimmune pancreatitis), or AP induced by special causes such as malignant tumors, trauma, or drugs.
3. Complicated with severe hepatobiliary diseases.

### Reagents

Caerulein was purchased from Merck (Darmstadt, Germany). Lentiviral vectors for NOX4 overexpression, NOX4 knockdown, and NCOA4 knockdown, including plasmids expressing NOX4, shNOX4, and shNCOA4, respectively, were obtained from GeneChem (Shanghai, China). The miR-25-3p mimics and antagomirs were synthesized by RiboBio (Guangzhou, China). Antibodies for Western blotting were acquired from Proteintech (Wuhan, China).

### Animals

Male C57BL/6J mice (6 weeks old) were obtained from Beijing Vital River Laboratory Animal Technology Co., Ltd. All animal procedures were approved by the Institutional Animal Care and Use Committee. Genomic DNA was extracted from tail biopsies for genotyping. To generate NOX4-overexpressing, NOX4-knockdown and NCOA4-knockdown mice, a tail vein injection of lentivirus encoding NOX4, shNOX4 or shNCOA4 was administered. miRNAs were overexpressed or knocked down through tail vein injection of chemically synthesized miRNA mimics or antagomirs. The dosage was administered at 8-10 nmol per mouse per session (approximately 2-3 mg/ kg, calculated based on a mouse weight of 25 g) on Days 0, 2, and 4. Samples were collected 48-72 hours after the final treatment. For pharmacological interventions, the mice received intravenous injections of erastin (15 mg/ kg) or ferrostatin-1 (Fer-1; 10 mg/ kg) one hour before the first caerulein administration.

### Establishment of the AP-LI animal model

After one week of acclimatization, the mice were fasted for 12 hours with free access to water. An AP-LI animal model was induced by twelve hourly intraperitoneal injections of caerulein (100 μg/kg), followed by a single intraperitoneal injection of lipopolysaccharide (LPS; 10 mg/ kg) 15 minutes after the final caerulein dose.^[21,22]^ The control mice received an equivalent volume of 0.9% saline. All the animals had free access to food and water following the injection. The mice were euthanized by cervical dislocation 12 hours after the final injection. Plasma, pancreatic tissue, and lung tissue samples were collected and stored at -80 °C for subsequent analysis.

### Cell culture, treatments and intervention

Lung tissues free of blood contamination were excised, and trypsin was introduced *via* the bronchial tree to perform enzymatic digestion of the lung tissue. AT2 cells were isolated using discontinuous density gradient centrifugation with either Ficoll or Percoll. The isolated cells were then resuspended in complete DMEM/F12 medium at a concentration of 1 × 10^6^ cells/mL and seeded onto type I collagen-coated plates at a density of 4 × 10^5^ cells/cm^2^. The cells were cultured at 37 °C in a 5% CO_2_ incubator for 24 hours prior to experimentation. For the miRNA overexpression and knockdown experiments, cells were seeded into 6-well plates at a density of 2 × 10^5^ to 4 × 10^5^ cells per well. When the cells reached 70%-90% confluence, the culture medium was replaced, and a total volume of 250 μL containing RNAiMAX and miRNA mimics (final concentration: 25-50 nmol/L) or antagomir (final concentration: 50-100 nmol/L) at a 1:1 volume ratio was evenly added to each well. For gene overexpression or knockdown *via* lentiviral transduction, 12-well plates were used with 1 × 10^5^ to 2 × 10^5^ cells seeded per well. When the cell confluence reached 50%-70%, a mixture of virus and polybrene was added. Following incubation at 37 °C and 5% CO_2_ for 6-8 hours, the culture medium was replaced, and puromycin was applied for antibiotic selection at a concentration of 1-3 μg/mL, starting 48 hours post-transduction and continuing for 5 days. The transduction efficiency was evaluated 24 hours after transduction, after which total RNA and protein were extracted. Bafilomycin A1 was dissolved in DMSO to prepare a 100 nmol/L working solution and applied to cultured cells for 6 hours. U0126, a selective MEK1/2 inhibitor, was similarly dissolved in DMSO to yield a 10 μmol/L working solution and administered to cells for 2 hours under standard *in vitro* conditions.

### Hematoxylin-eosin (H&E) and masson’s trichrome staining

The tissues were fixed in 4% paraformaldehyde for 24 hours, dehydrated, and embedded in paraffin. The sections were deparaffinized with xylene and a graded ethanol series. For H&E staining, the sections were stained with hematoxylin, rinsed, differentiated with acid alcohol, and blued in ammonia water, followed by eosin counterstaining. For Masson staining, the sections were sequentially stained with bright cresyl red/acid fuchsin and bright green. All the slides were dehydrated, cleared with xylene, and mounted.

### Lentiviral vector construction and in vivo administration

Recombinant lentiviral vectors for NOX4 overexpression (Lv_NOX4) and NOX4 knockdown (Lv_shNOX4) were constructed using a third-generation lentiviral backbone under the control of the CMV promoter (GeneChem, Shanghai, China). Short hairpin RNA (shRNA) sequences targeting mouse NOX4 were designed based on validated target sequences (Table S1), and a nontargeting shRNA was used as a negative control (Lv_NC). Lentiviral particles were packaged in HEK293T cells using standard packaging plasmids and concentrated by ultracentrifugation. Viral titers were determined by serial dilution and expressed as transducing units per milliliter (TU/mL). The final titers of the lentiviral preparations used for the *in vivo* experiments ranged from 1 × 10^8^ to 5 × 10^8^ TU/mL. For *in vivo* gene modulation, mice were randomly assigned to experimental groups and received lentiviral administration *via* tail vein injection at a dose of 100 μL per mouse (approximately 1 × 10^7^ TU per animal), as previously described. Lentiviral delivery was performed 72 hours prior to acute pancreatitis induction to allow sufficient transgene expression or gene silence. The mice were subsequently subjected to AP-LI modeling and sacrificed at the indicated time points for tissue collection and analysis.

### Histopathological analysis

Six randomly selected fields per section were scored for lung injury using a standardized histopathological scoring system (Table S2).^[23]^ Each criterion was graded on a scale of 0-5, and the final score was the average of six measurements. Pulmonary edema was assessed by calculating the lung wet-to-dry (W/D) weight ratio. Fresh tissues were weighed (wet weight), dried at 80 °C for 48 hours, and weighed again (dry weight). The W/ D ratio was calculated accordingly. Histopathological injury of the pancreas was evaluated using the Schmidt scoring system, which semi-quantitatively assesses pancreatic damage based on four parameters: interstitial edema, inflammatory cell infiltration, acinar cell necrosis, and hemorrhage/ fat necrosis. Each parameter was scored on a scale of 0-3 according to severity, and the total histological score was calculated as the sum of the individual parameter scores (Table S3). For each sample, at least five randomly selected non-overlapping high-power fields (HPFs) were examined under light microscopy (200 × magnification). All histological evaluations were performed independently by two experienced investigators who were blinded to the experimental groups, and the final score for each sample was calculated as the average of the two assessments.^[24]^

### Transmission electron microscopy (TEM)

A 10 μL aliquot of the exosome suspension was placed on a copper grid and allowed to settle for 1 minute. Excess fluid was blotted, and the grids were stained with 10 μL of uranyl acetate for 1 minute, followed by air drying. Exosomes were visualized using a TEM at 100 kV. For ultrastructural analysis, fresh lung tissues were fixed with glutaraldehyde and osmium tetroxide, dehydrated in graded ethanol, and embedded in epoxy resin. Ultrathin sections (80 nm) were stained with uranyl acetate and lead citrate. Mitochondrial and AT2 cell morphology was examined using a Hitachi H-7100 transmission electron microscope.

For quantitative assessments of ferroptosis-associated mitochondrial ultrastructural damage *via* TEM, six mice per experimental group were randomly selected. At least ten nonredundant cellular cross-sections were sampled from each animal. Within each selected cell, three non-overlapping fields of view were randomly acquired at uniform magnification, and ten morphologically intact mitochondria were systematically identified and analyzed per field. All image acquisition, selection, and morphometric analyses were performed in a blind manner to minimize observer bias. The ferroptosis-like mitochondrial ratio was calculated as the percentage of ferroptosis-like mitochondria among total mitochondria. A mitochondrion was classified as “ferroptosis-like” if it met at least two of the following criteria: (1) marked reduction or complete loss of cristae; (2) significant reduction in the mitochondrial cross-sectional area; (3) increased outer membrane electron density; (4) discontinuity or rupture of the outer mitochondrial membrane.

### Exosome extraction and purification

Frozen plasma samples were thawed at 37 °C. After centrifugation at 2000×*g* for 30 minutes at 4 °C, the supernatants were centrifuged at 10,000×*g* for 45 minutes to remove large vesicles. The samples were filtered through a 0.45 μm membrane and ultracentrifuged at 100,000×*g* for 70 minutes at 4 °C. The pellet was subsequently washed in cold phosphate-buffered saline (PBS) and centrifuged under the same conditions. The final exosome pellet was resuspended in 200 μL of PBS and stored at -80 °C for subsequent analyses.

### Exosome particle size analysis, quantification and normalization

A 10 μL aliquot of exosomes was diluted to 30 μL for measurement using a NanoFCM system. Calibration was performed with standard particles to ensure accuracy. The particle size distribution and concentration were recorded. To ensure consistent exosome administration across the experimental groups, the exosome dosage was normalized based on NanoFCM-based particle counting. Following isolation and purification, exosome suspensions were quantified using NanoFCM, and equal numbers of exosomal particles were administered in each experiment.

### Fluorescence labeling and nano-flow cytometry of exosomes

Exosomes (30 μL) were diluted to 90 μL in buffer. Each 30 μL aliquot was incubated with 20 μL of fluorescently labeled antibodies against CD9, CD63, or CD81 at 37 °C for 30 minutes in the dark. The samples were subsequently washed twice via ultracentrifugation at 110,000×*g* for 70 minutes at 4 °C in precooled PBS. The final pellet was resuspended in 50 μL of PBS. Surface marker expression was analyzed by nanoflow cytometry following system calibration.

### Construction of engineered exosomes and in vivo administration

The human embryonic kidney cell line HEK293T (ATCC) was utilized for the generation of engineered exosomes. Cells were cultured in Dulbecco’s modified Eagle’s medium (DMEM) supplemented with 10% fetal bovine serum (FBS) and 1% penicillin-streptomycin solution and maintained in a humidified incubator at 37 °C under 5% CO_2_. To minimize contamination from exogenous exosomes, FBS was pretreated by ultracentrifugation at 100,000×*g* for 16 hours to prepare exosome-depleted FBS prior to experimental use. When the HEK293T cells reached 70%-80% confluence, the miR-25-3p mimic was transfected using Lipofectamine 3000 transfection reagent (catalog No. L3000008, Invitrogen, Thermo Fisher Scientific, Waltham, MA, USA) according to the manufacturer’s protocol. After 6-8 hours of transfection, the medium was replaced with fresh DMEM containing exosome-depleted FBS. The transfection efficiency was confirmed *via* RT-qPCR by measuring the intracellular levels of miR-25-3p, with control cells receiving a negative control mimic. Forty-eight hours post-transfection, the cell culture supernatants were collected, and the exosomes were isolated through differential centrifugation. The final exosome pellet was resuspended in an appropriate volume of PBS and stored at -80 °C until further use. The engineered exosomes obtained were identified by TEM, NanoFCM analysis and Western blotting. After the AP-LI models were induced, the mice were administered engineered exosomes *via* tail vein injection at a dose of 1 × 10^10^ particles per mouse. Negative control animals received mimic negative control (NC) with the same particle quantity. Exosome administration was performed once every 6 hours for two consecutive doses. Mice were euthanized 6 hours after the final treatment, and lung tissues and other relevant samples were harvested for subsequent histological, molecular, and biochemical analyses.

### Western blot

Cells and tissues were lysed in RIPA buffer supplemented with protease and phosphatase inhibitors. The proteins were separated by SDS-PAGE and transferred onto PVDF membranes. The membranes were blocked with 5% skim milk for 1 hour at room temperature and incubated overnight at 4 °C with primary antibodies (1:1000, Proteintech) followed by HRP-conjugated secondary antibodies (1:5000, Proteintech) for 1 hour at room temperature. The protein bands were visualized using an enhanced chemiluminescence (ECL) kit (Bio‑Rad Laboratories, Hercules, CA, USA) and imaged with an Odyssey Imaging System (LI‑COR Biosciences, Lincoln, NE, USA). β-Actin was used as the internal control. The band intensities were quantified using ImageJ software (National Institutes of Health, Bethesda, MD, USA) based on three independent biological replicates.

### Chromatin immunoprecipitation (ChIP)

The binding sites of hypoxia inducible factor 1 subunit alpha (HIF-1α) and NCOA4 were computationally predicted using the UCSC Genome Browser and JASPAR transcription factor binding profile databases. ChIP-qPCR was employed to experimentally validate HIF-1α occupancy at promoter-proximal regions in AT2 cells. Briefly, cells were crosslinked with 1% methanol-free formaldehyde for 10 minutes at room temperature and the reaction was quenched with 125 mmol/L glycine for 5 minutes. Following three washes with ice-cold PBS, chromatin was isolated, sonicated to achieve fragment sizes of 200-500 bp, and clarified by centrifugation. An aliquot of clarified chromatin was diluted 1:10 in ChIP dilution buffer supplemented with protease inhibitors; 1% of this diluted sample was retained as the “input” control. The remaining chromatin was pre-cleared with protein A/ G magnetic beads and subsequently incubated overnight at 4 °C with either anti-HIF-1α antibody (Proteintech, Wuhan, China) or isotype-matched IgG control antibody. Immunocomplexes were captured using protein A/G magnetic beads, followed by sequential washes with low-salt, high-salt, LiCl, and TE buffers. DNA was eluted from the beads, and crosslinks were reversed by incubation at 65 °C overnight after treatment with RNase A and proteinase K. Purified DNA was quantified by SYBR Green-based qPCR using primers targeting promoter-proximal regions. Enrichment was calculated as percentage of input [% input = 100 × 2^(Ct_input - log2(100) - Ct_IP)]. All ChIP-qPCR data represent the mean ± SD from three biologically independent experiments.

### RNA isolation, reverse transcription, and qPCR

Total RNA was extracted using TRIzol reagent (Thermo Fisher, Shanghai, China). Reverse transcription was performed using a commercial kit (Thermo Fisher, Shanghai, China), and qPCR was conducted using SYBR Green (Solarbio, Beijing, China) on an Applied Biosystems 7500 system. U6 was used as the reference for miR-25-3p, and GAPDH served as the reference for mRNA targets, including NOX4. The primer sequences are listed in Supplementary Table S4.

### Argonaute-2 RNA immunoprecipitation (AGO2-RIP)

AGO2-RIP was performed in primary mouse AT2 cells using an anti-AGO2 antibody (Proteintech, Cat# 67934-1-Ig) to capture AGO2-containing RNA-induced silencing complex (RISC). Cells were lysed on ice in RIP lysis buffer supplemented with protease inhibitor cocktail and RNase inhibitor. The lysates were clarified by centrifugation, and 1% of each lysate was reserved as input. The remaining lysates were precleared with protein A/ G magnetic beads and then incubated with anti-AGO2 antibody (Proteintech, 67934-1-Ig) or isotype-matched mouse IgG at 4 °C with rotation. Immune complexes were captured with Protein A/G magnetic beads and were subsequently washed thoroughly at 4 °C. RNA was released by proteinase K digestion and extracted for downstream RT-qPCR analysis. For qPCR quantification, 1% of the total lysate was reserved as input. RT-qPCR was performed to quantify target RNA in the AGO2 and IgG immunoprecipitated, and enrichment was expressed as % input after the 1% input Ct for dilution was corrected [Ct_adjusted = Ct_input - log2(100)].

### Luciferase reporter assay

For the miR-25-3p/NOX4 assay, WT and MUT NOX4 the 3′ untranslated region (3′ UTR) reporter plasmids were cotransfected with the miR-25-3p mimic or NC mimic. For the HIF-1α/NCOA4 assay, WT and MUT NCOA4 promoter reporter plasmids were cotransfected with the HIF-1α overexpression vector or control vector. After 48 hours, luciferase activity was measured using the Dual-Luciferase Reporter Assay System (Promega, WI, USA). Luciferase activity was normalized to Renilla luciferase activity.

### Oxidative stress assays

Fe^2+^, glutathione (GSH) , H_2_O_2_, and malondialdehyde (MDA) levels in lung tissues were measured using commercial assay kits (Wuhan Gill Biotechnology, China) in accordance with the manufacturer’s protocols.

### Immunohistochemistry (IHC)

Paraffin-embedded tissue sections were deparaffinized, rehydrated, and treated with 3% H_2_O_2_ to quench endogenous peroxidase activity. After blocking with 3% BSA for 30 minutes, the sections were incubated overnight at 4 °C with primary antibodies against 4-hydroxynonenal (4-HNE) and myeloperoxidase (MPO) (1:200; Proteintech). After washing, the sections were incubated with HRP-conjugated secondary antibodies (1:200; Proteintech) for 1 hour. DAB reagent (Abcam) was used for signal development, and the sections were counterstained with hematoxylin, dehydrated, cleared, and mounted. Positive staining was visualized under a light microscope.

### Cell viability assay

AT2 cells were seeded into 96-well plates at a density of 8 × 10^3^ cells/well. When the cell confluence reached approximately 80%, 10 μL of cell counting kit-8 (CCK-8) reagent (NCM Biotech, Suzhou, China) was added to each well, and the cells were incubated at 37 °C for 1 hour. The absorbance was measured at 450 nm using a microplate reader.

### ROS measurement

Intracellular ROS levels were assessed using a DCFH-DA (ROS detection) kit. The cells were incubated with the fluorescent probe according to the instructions of the kit, and the fluorescence intensity was analyzed under a fluorescence microscope. Background subtraction was performed using ImageJ. DCF fluorescence intensity was quantified from region-of-interest (ROI) measurements, and the ROS signal was normalized per nucleus as follows: (integrated fluorescence intensity - background intensity × ROI area) / number of nuclei.

### Mitochondrial membrane potential (MMP) assay

Cells were stained with 2.5 μg/mL JC-1 dye for 15 minutes, equilibrated for 10 minutes at room temperature, and visualized under a fluorescence microscope (Carl Zeiss, Germany) at 529 nm (green) and 590 nm (red). The MMP was evaluated based on the red/ green fluorescence ratio, which was quantified in ImageJ after background subtraction. For each independent experiment, at least 6 cells were analyzed per condition.

### Dihydroethidium (DHE) fluorescence staining

Fresh frozen tissue sections were equilibrated to room temperature for 20 minutes. Nuclei were stained with DAPI (1:500 dilution, 3 minutes, in the dark), followed by rinsing. The sections were then incubated with DHE working solution (1:400 dilution in TBST) for 1 hour in the dark, washed, and mounted with anti-fade reagent. The distribution of ROS was observed under a fluorescence microscope. For quantification, DHE fluorescence was analyzed using Fiji/ ImageJ. A tissue mask was generated to exclude alveolar airspaces, and the fluorescence intensity was measured within the tissue area. Background fluorescence was obtained from cell-free regions on the same section and subtracted to yield the corrected mean fluorescence intensity (MFI) (MFI_corrected = MFI_tissue - MFI_background). For each animal, 5 randomly selected fields from at least two sections were analyzed and averaged to obtain one value per animal.

### FerroOrange staining

Cells cultured in fluorescent dishes or 6-well plates were washed with HBSS or serum-free medium and incubated with 1 μmol/L FerroOrange working solution for 30-60 minutes at 37 °C. Intracellular Fe^2+^ was visualized using a fluorescence microscope. Background subtraction was performed using ImageJ. FerroOrange fluorescence intensity was quantified from ROI measurements, and the Fe^2+^ signal was normalized per nucleus as follows: (integrated fluorescence intensity - background intensity × ROI area) / number of nuclei.

### PKH26 exosome labeling and cellular uptake

Exosomes were labeled with 100 μmol/L PKH26 dye and incubated for 10 minutes in the dark. After the samples were washed with PBS and filtered through a 10 kDa centrifugal filter (3000×*g*, 10 minutes), the final volume was adjusted to 50-100 μL. Approximately 20 μg of labeled exosomes were added to AT2 cell cultures and incubated for 4-8 hours at 37 °C. Uptake was visualized under a fluorescence microscope.

### Autophagy flux assay

AT2 cells were transfected with tandem mRFP-GFP-LC3 lentivirus (GeneChem, Shanghai). This construct enabled visualization of autophagosomes (yellow puncta: GFP+RFP+) and autolysosomes (red puncta: RFP only). After treatment, the cells were imaged using a fluorescence microscope (Carl Zeiss, Germany), and autophagic flux was analyzed using the ImageXpress Pico system by calculating the red/yellow puncta ratio.

### Statistical analysis

The statistical analyses in this study were performed using GraphPad Prism software (version 9.5.1). For comparisons between two groups, a two-tailed unpaired Student’s *t* test was applied. For comparisons involving more than two groups, one-way analysis of variance (ANOVA) followed by Tukey’s multiple comparisons test was used. Correlation analyses were performed using Pearson’s correlation coefficient. All experiments were repeated at least three times independently, and the experimental data conformed to standard statistical principles and practices. The sample sizes (*n*) for each experiment are specified in the corresponding figure legends. A *P* value < 0.05 was considered to indicate statistical significance (^*^P < 0.05, ^**^P < 0.01, ^***^P < 0.001, ^****^P < 0.0001, ns not significant).

### Randomization, blinding, and sample size

Animals were randomly assigned to experimental groups after successful model establishment. Investigators responsible for histological assessment, fluorescence quantification, and data analysis were blinded to group allocation. Sample sizes were determined based on preliminary experiments, previous studies using similar animal models, and ethical considerations to minimize animal use. No formal a priori power calculation was performed.

## Results

### Plasma exosomes from AP-LI animal models aggravate AP-LI

To investigate the role of plasma exosomes in AP-LI, we established an AP-LI model in C57BL/6J mice by intraperitoneal injection of caerulein (Figure 1A). Blood samples were collected, and plasma exosomes were isolated by differential centrifugation (Figure 1B). H&E staining of pancreatic tissue showed decreased acinar nuclear volume, chromatin condensation, cytoplasmic vacuolization, and partial destruction of acinar architecture in AP-LI mice. Lung tissue from AP-LI mice showed extensive inflammatory exudation and edema within the alveolar spaces, alveolar collapse and atrophy, and marked disruption of alveolar structure (Figure 1C). Serum amylase and lipase levels were also significantly increased in AP-LI mice compared with control mice, confirming successful model establishment (Figure 1D). Transmission electron microscopy revealed the typical round or cup-shaped morphology of exosomes in both groups; however, exosomes from AP-LI mice showed a larger diameter, mild collapse, and partial membrane damage (Figure 1E). Nanoparticle tracking analysis showed that exosomes from control mice had an average diameter of 78.2 nm and a concentration of 3.10 × 10^9^ particles/mL, whereas exosomes from AP-LI mice had an average diameter of 82.3 nm and a significantly lower concentration of 1.85 × 10^9^ particles/mL (Figure 1F). Nanoflow cytometry showed no significant difference in the percentages of CD9, CD63, and CD81-positive particles between AP-LI and control plasma exosomes (Figure 1G). Western blotting further confirmed the exosomal identity of the isolates through the positive markers TSG101 and CD63 and the negative marker calnexin (Figure 1H). Primary AT2 cells isolated from AP-LI lungs were incubated with PKH26-labeled exosomes, and fluorescence microscopy demonstrated efficient cellular uptake (Figure 1I). To assess the biological effect of plasma exosomes, equal numbers of exosomal particles from control or AP-LI mice were injected into separate groups of AP-LI mice. Compared with AP-LI mice without exosome injection, AP_exo-injected mice showed more severe alveolar inflammation, tissue edema, vascular congestion, and alveolar collapse. Histological injury scores, W/ D weight ratios, and serum levels of SP-D, KL-6, IL-1β, and TNF-α were all significantly increased in AP_exo-injected mice. In contrast, ctrl_exo injection did not significantly alter these parameters compared with control mice (Supplementary Figure S1A-F). These findings indicate that plasma exosomes from AP-LI mice undergo both morphological and functional alterations, are taken up by AT2 cells, and aggravate lung injury.

**Figure 1:**
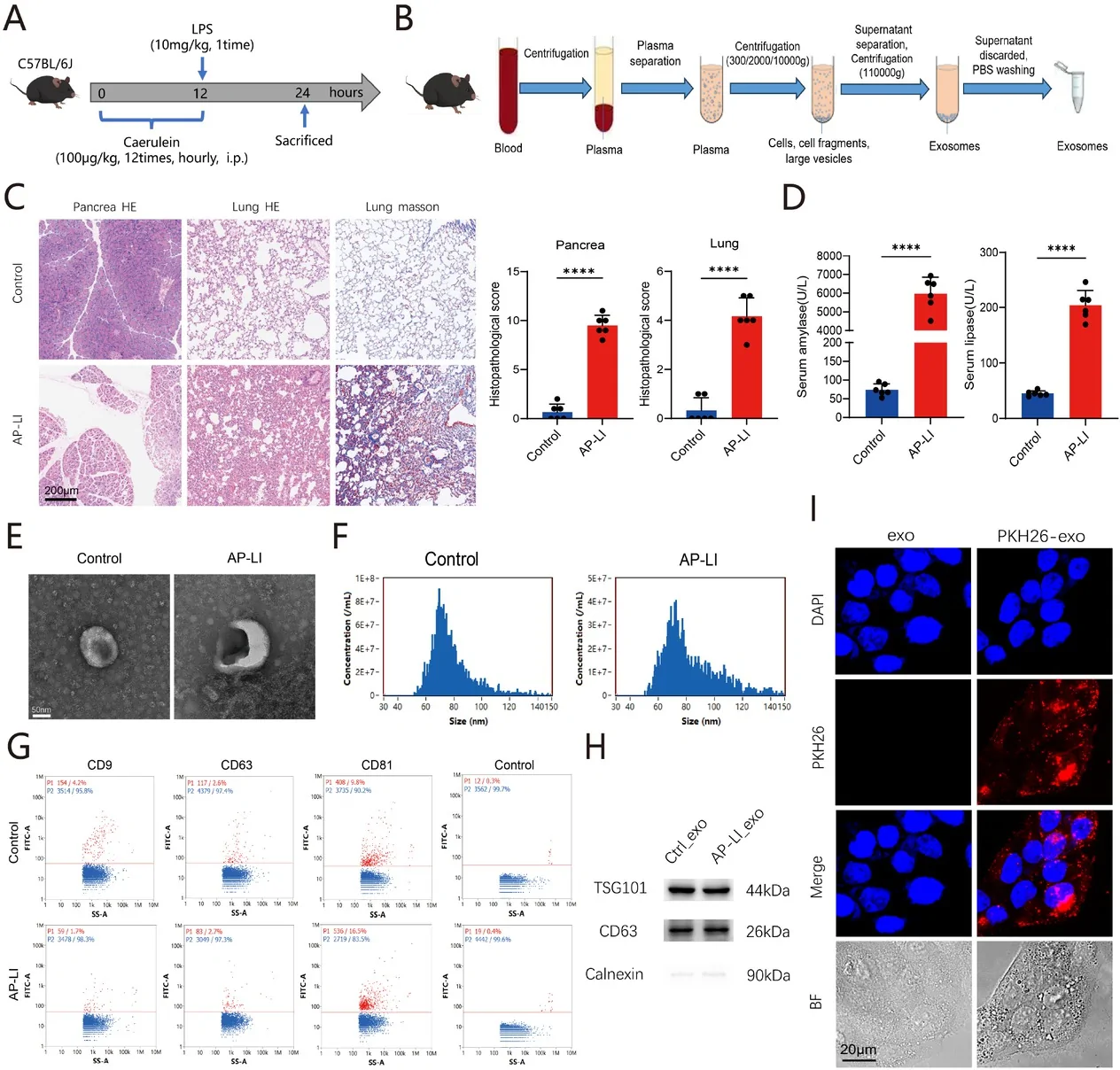
Plasma exosomes from AP-LI animal models aggravate AP-LI. (A) Schematic illustration of the experimental mouse model induced by caerulein injection. (B) Plasma exosome extraction process. (C) Representative H&E and Masson staining images of pancreatic tissue and histopathological scores of pancreatic and lung tissue sections. Scale bar = 200 μm. (D) Serum amylase and lipase levels in control and AP-LI mouse plasma. (E) Representative TEM images of plasma exosomes. Scale bar = 50 nm. (F) Particle size and concentration of plasma exosomes. (G) Surface marker analysis (CD9, CD63, and CD81) of plasma exosomes. (H) Protein expression levels of exosomal markers (including TSG101, CD63 and calnexin). (I) PKH26-labeled plasma exosomes internalized by AT2 cells. Scale bar = 20 μm. The data are presented as the mean ± SD (*n* = 6). Statistical significance was determined by one-way ANOVA followed by Tukey’s multiple comparisons test. ^*^P < 0.05, ^**^P < 0.01, ^***^P < 0.001, ^****^P < 0.0001, ns not significant. H&E: hematoxylin-eosin; AP-LI: acute pancreatitis-associated lung injury; AT2: alveolar type II epithelial; TEM: transmission electron microscopy.

### MiR-25-3p is downregulated in plasma exosomes from AP-LI animal models

Because exosomes carry diverse bioactive molecules, we next sought to identify exosomal components involved in AP-LI. Small RNA sequencing was performed on plasma exosomes isolated from control and AP-LI mice. A total of 86 differentially expressed miRNAs were identified (*P* < 0.05), including 42 upregulated and 44 downregulated miRNAs in AP-LI plasma exosomes (Figure 2A). Both the composition and abundance of exosomal miRNAs differed markedly between the two groups (Figure 2B-D). Based on differential expression analysis, the 10 most altered miRNAs were selected for validation (Figure 2E), and their expression levels were quantified by qPCR in plasma exosomes from control and AP-LI mice (Figure 2F). Among these candidates, miR-25-3p was the most significantly downregulated and was therefore selected for further study. Gene Ontology (GO) and Kyoto Encyclopedia of Genes and Genomes (KEGG) analyses of the predicted target genes of the differentially expressed miRNAs showed enrichment in protein binding, metal ion binding, cytoplasm, membrane components, and several oxidative stress-related pathways, including Wnt/beta-catenin and FoxO signaling (Figure 2G, H and Supplementary Figure S2A B). These data suggest that plasma exosomal miR-25-3p may participate in the regulation of oxidative stress and ferroptosis during AP-LI.

**Figure 2:**
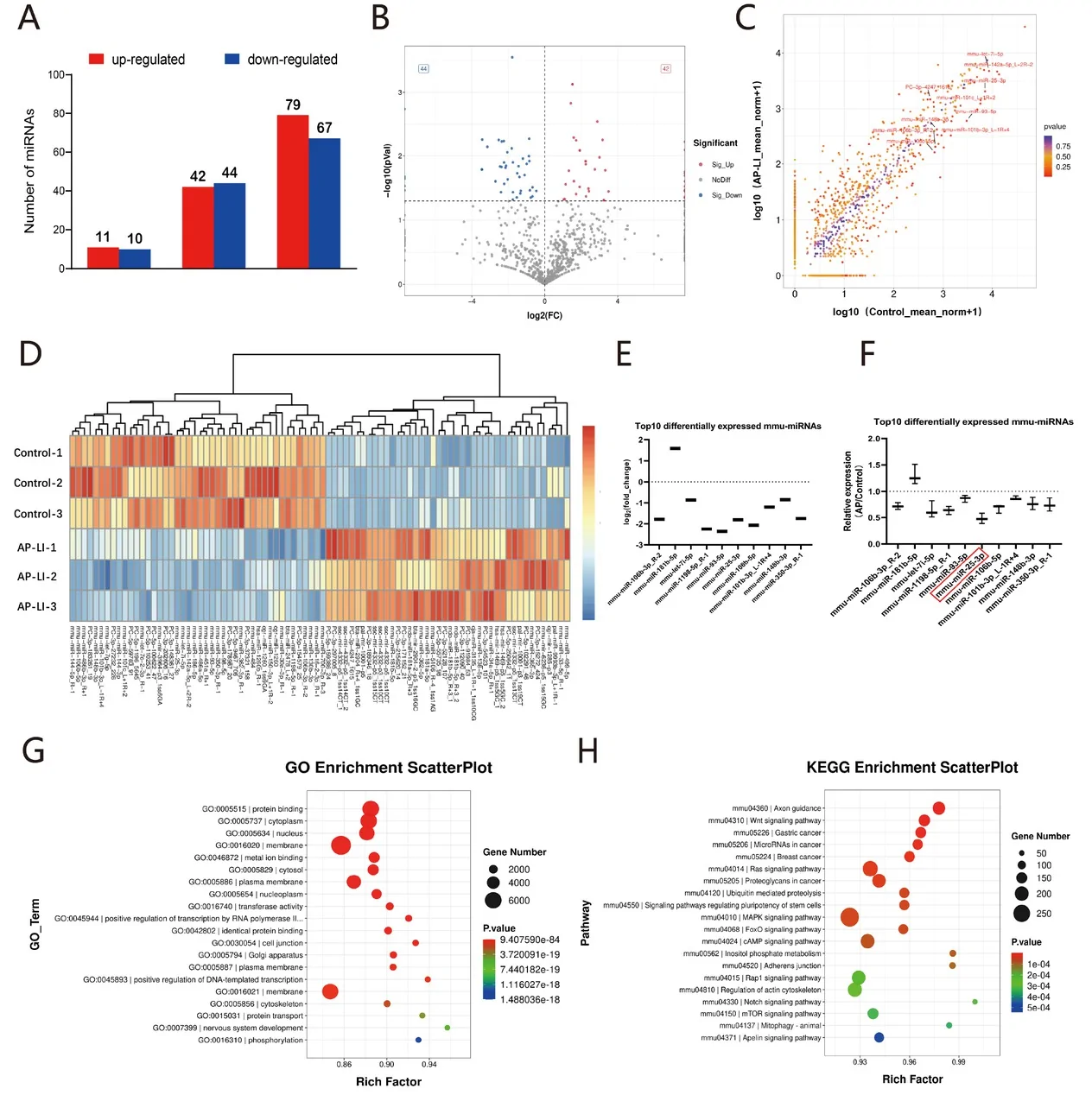
miR-25-3p is downregulated in plasma exosomes from AP-LI animal models. (A) Bar chart of differentially expressed miRNAs. (B) Volcano plot of the distribution of miRNAs. (C) Scatter plot of the distribution of miRNAs. (D) Heatmaps showing the expression profiles of altered miRNAs. (E) Top 10 differentially expressed miRNAs identified from sequencing. (F) Validation of miRNA expression levels using qPCR. (G) GO enrichment scatterplot of the miRNA sequencing result. (H) KEGG enrichment scatterplot of the miRNA sequencing result. AP-LI: acute pancreatitis-associated lung injury; GO: Gene Ontology; KEGG: Kyoto Encyclopedia of Genes and Genomes.

Plasma exosomes are derived from multiple tissues. To determine whether the reduction in plasma exosomal miR-25-3p could, at least in part, originate from injured pancreatic tissue, we isolated exosomes from pancreatic tissue and from primary pancreatic acinar cells in control and AP-LI mice. qPCR showed that miR-25-3p levels were significantly lower in pancreatic tissue-derived exosomes and acinar cell-derived exosomes from AP-LI mice than in the corresponding control samples (Supplementary Figure S3A, B). Moreover, AP-LI mice that received intravenous injection of pancreatic tissue-derived exosomes or primary pancreatic acinar cell-derived exosomes from AP-LI donors displayed more severe lung injury (Supplementary Figure S3C, D). These findings suggest that injured pancreatic tissue is an important source of miR-25-3p-deficient exosomes in AP-LI and that these exosomes may aggravate lung injury after entering the circulation.

To further test whether exosomal deliver y of miR-25-3p confers lung protection, we generated engineered exosomes overexpressing miR-25-3p and performed systematic characterization and validation (Supplementary Figure S4A-D). These engineered exosomes were administered intravenously to AP-LI mice, whereas the negative control group received equal numbers of empty-vector engineered exosomes. H&E staining and multiple indices of lung injury showed that treatment with miR-25-3p-enriched exosomes markedly attenuated lung injury, whereas the negative control exosomes produced no significant benefit. In contrast, pancreatic injury, serum amylase, and serum lipase levels did not differ significantly between the negative control and miR-25-3p-engineered exosome groups (Supplementary Figure S4E-J). Together, these results support a protective role for exosome-delivered miR-25-3p in AP-LI and suggest that this intervention acts primarily at the level of the lung rather than the pancreas.

### Plasma exosomal miR-25-3p alleviates AP-LI by inhibiting ferroptosis in vivo and in vitro

Given that the bioinformatic analyses implicated oxidative stress and ferroptosis, we next examined whether plasma exosomal miR-25-3p regulates ferroptosis during AP-LI. *In vivo*, effective knockdown and overexpression of miR-25-3p were achieved using a miR-25-3p antagomir and mimic, respectively (Supplementary Figure S6A, B). *In vitro*, primary AT2 cells isolated from AP-LI mice were transfected with a miR-25-3p mimic or inhibitor, and transfection efficiency was confirmed by qPCR (Supplementary Figure S6C, D). To verify that miR-25-3p acts as a functional effector of plasma exosomes, miR-25-3p inhibitors or mimics were administered to AP-LI mice receiving AP_exos. miR-25-3p inhibition aggravated AP_exo-associated lung injury and ferroptosis, whereas miR-25-3p overexpression had the opposite effect (Figure 3A and Supplementary Figure S6E-I). Compared with control mice, AP-LI mice showed increased Fe^2+^ accumulation, enhanced lipid peroxidation, and altered expression of ferroptosis-related proteins in lung tissue. miR-25-3p knockdown further intensified these ferroptotic changes and worsened lung injury, whereas miR-25-3p overexpression reduced ferroptosis-associated markers and alleviated tissue damage (Figure 3B and Supplementary Figure S6J-O). Fluorescence assays further showed that miR-25-3p knockdown increased ROS and Fe^2+^ levels and decreased MMP; these effects were attenuated by the ferroptosis inhibitor Fer-1. Conversely, miR-25-3p overexpression reduced ROS and Fe^2+^ accumulation and restored MMP, and these protective effects were reversed by the ferroptosis inducer erastin (Figure 3C-E).

**Figure 3:**
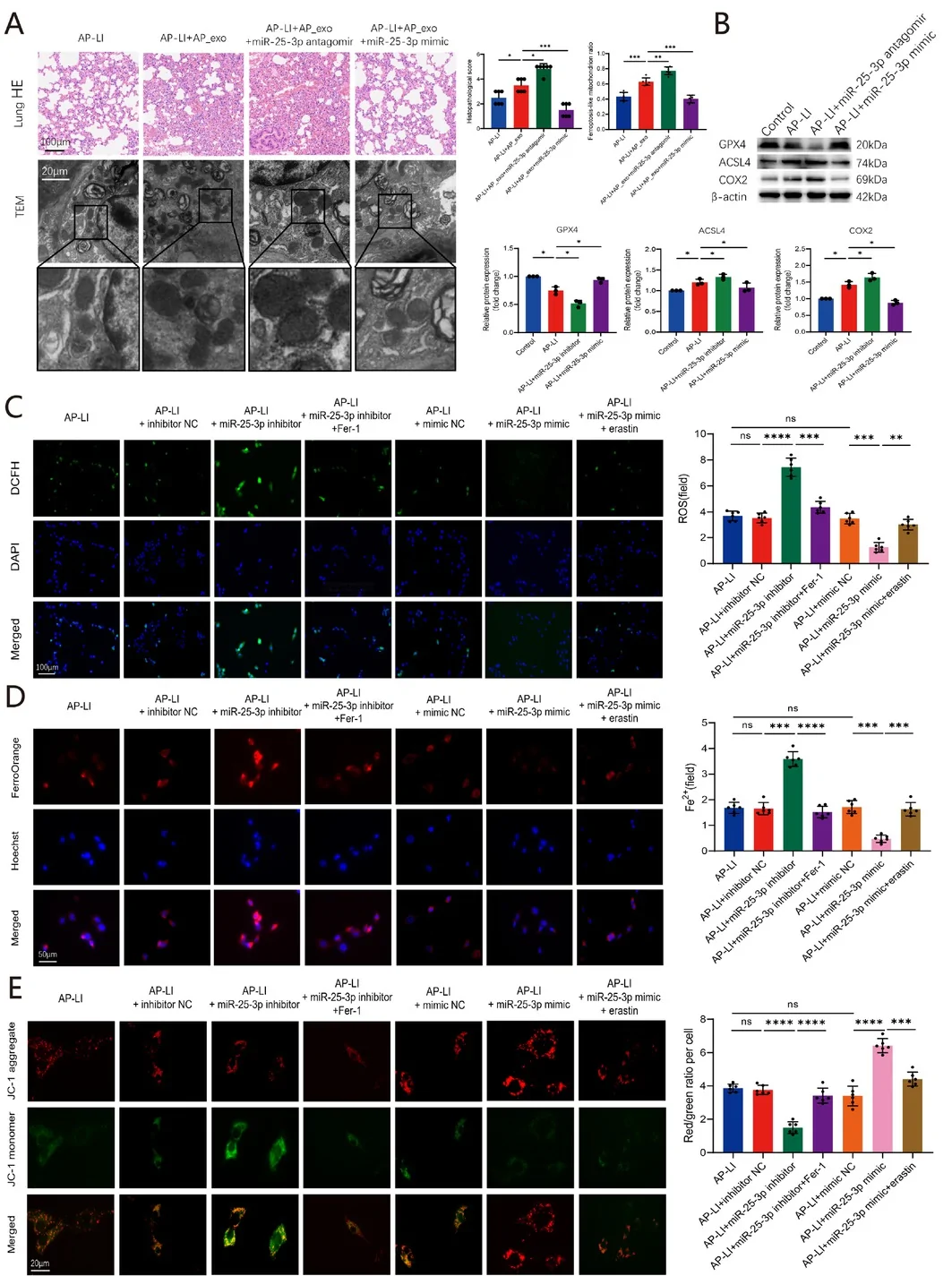
Plasma exosomal miR-25-3p alleviates AP-LI by inhibiting ferroptosis *in vivo* and *in vitro*. (A) Representative H&E staining images of lung tissues and histopathological scores from AP-LI, AP-LI + AP_exo, AP-LI + AP_exo + miR-25-3p antagomir and AP-LI + AP_exo + miR-25-3p mimic groups. Scale bar = 200 μm. Representative TEM images of AT2 cells in lung tissues from AP-LI, AP-LI + AP_exo, AP-LI + AP_exo + miR-25-3p antagomir and AP-LI + AP_exo + miR-25-3p mimic groups. Scale bar = 20 μm. (B) Representative Western blot images and quantitative analysis of GPX4, ACSL4, and COX2 protein expression in lung tissues from control, AP-LI, AP-LI + miR-25-3p antagomir and AP-LI + miR-25-3p mimic groups; β-actin served as a loading control. (C) ROS levels were assessed with DCFH/DAPI staining. Scale bar = 100 μm. (D) Fe^2+^ levels detected by FerroOrange staining. Scale bar = 50 μm. (E) Mitochondrial membrane potential was assessed using JC-1 staining. Scale bar = 25 μm. The data are presented as the mean ± SD (A, C-E: *n* = 6; B : *n* = 3). Statistical significance was determined by one-way ANOVA followed by Tukey’s multiple comparisons test. ^*^P < 0.05, ^**^P < 0.01, ^***^P < 0.001, ^****^P < 0.0001, ns not significant. H&E: hematoxylin-eosin; AT2: alveolar type II epithelial; GPX4: glutathione peroxidase 4; ACSL4: acyl-CoA synthetase long chain family member 4; COX2: cyclooxygenase-2; ROS: reactive oxygen species; MDA: malondialdehyde; GSH: glutathione; Fer-1: ferrostatin-1.

Consistent with these findings, miR-25-3p knockdown in AP-LI mice significantly increased the expression of key ferroptosis-associated proteins and elevated multiple biochemical markers of cellular injury in lung tissue (Supplementary Figure 5A, C-G). H&E staining and histopathological scoring further confirmed aggravated structural damage in the lung (Supplementary Figure S5B). Notably, these pathological and molecular alterations were effectively attenuated by coadministration of Fer-1, supporting a causal role for ferroptosis in AP-LI. Taken together, these results demonstrate that plasma exosomal miR-25-3p alleviates AP-LI, at least in part, by suppressing ferroptosis *in vivo* and *in vitro*.

**Figure 4:**
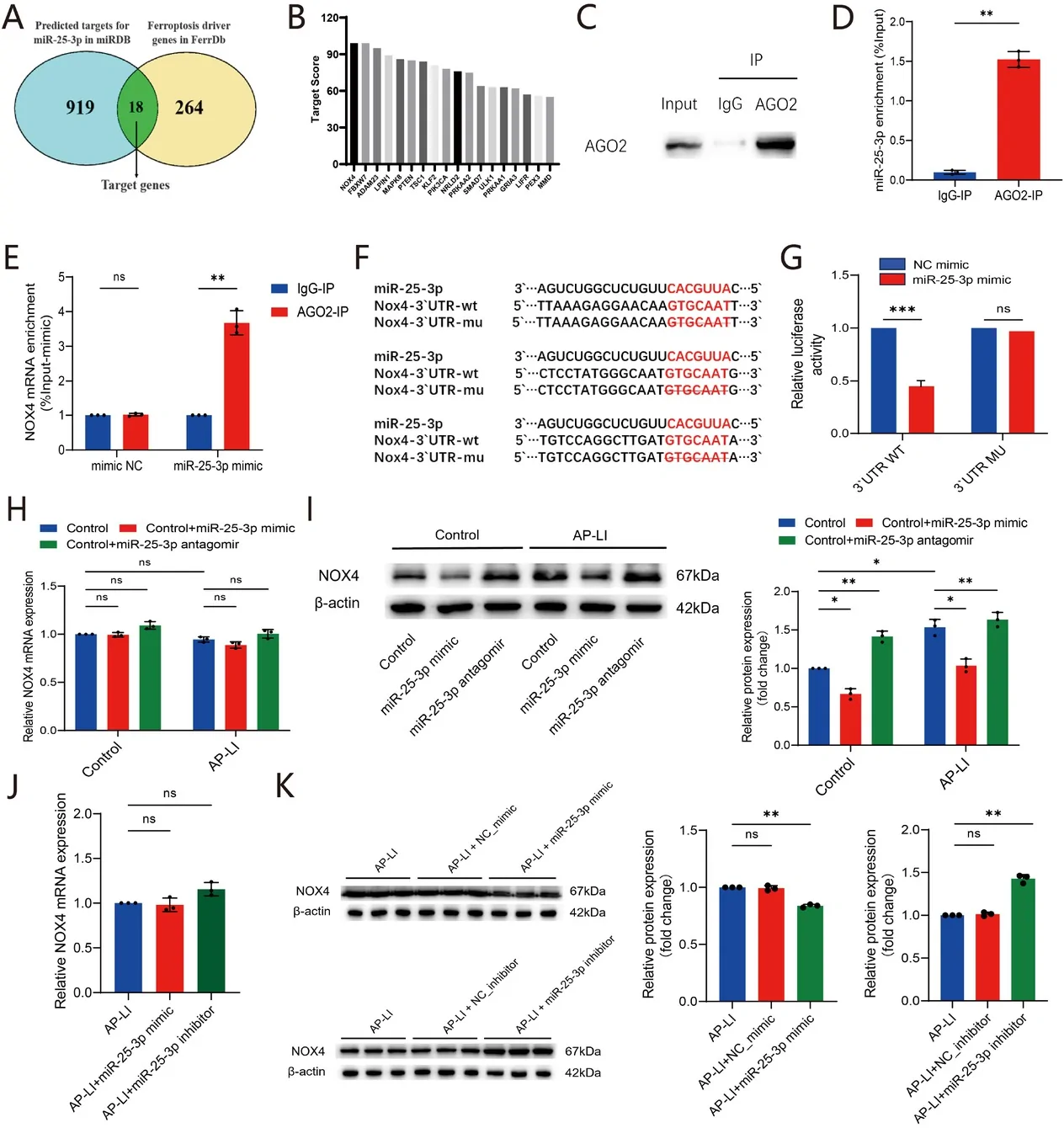
Plasma exosomal miR-25-3p alleviates AP-LI by negatively regulating NOX4. (A) Venn diagram of predicted miR-25-3p targets (miRDB) and ferroptosis-related genes (FerrDb). (B) Predicted target gene ranking based on the miRDB score. (C) RIP was performed in AT2 cells against IgG or AGO2. (D) miR-25-3p enrichment (% input) in IgG-IP and AGO2-IP. The data are presented as the mean ± SD (*n* = 3). Differences were analyzed using a two-tailed unpaired Student’s *t* test. (E) NOX4 mRNA relative enrichment in IgG-IP and AGO2-IP of mimic NC and miR-25-3p mimic. The data are presented as the mean ± SD (*n* = 3). Differences were analyzed using a two-tailed unpaired Student’s t test. (F, G) Luciferase activity assays showing the binding of miR-25-3p to the wild-type and mutant NOX4 3' UTR reporters. The data are presented as the mean ± SD (*n* = 3). Differences were analyzed using a two-tailed unpaired Student’s *t* test. (H, I) NOX4 mRNA and protein expression levels in lung tissues after miR-25-3p modulation; β-actin was used as a loading control. (J, K) NOX4 mRNA and protein expression levels in transfected AT2 cells. The data are presented as the mean ± SD (*n* = 3). Statistical significance was determined by one-way ANOVA followed by Tukey’s multiple comparisons test. ^*^P < 0.05, ^**^P < 0.01, ns not significant. AP-LI: acute pancreatitis-associated lung injury; NOX4: NADPH oxidase 4; AT2: alveolar type II epithelial; AGO2: argonaute-2; NC: negative control; 3' UTR: the 3' untranslated region.

**Figure 5:**
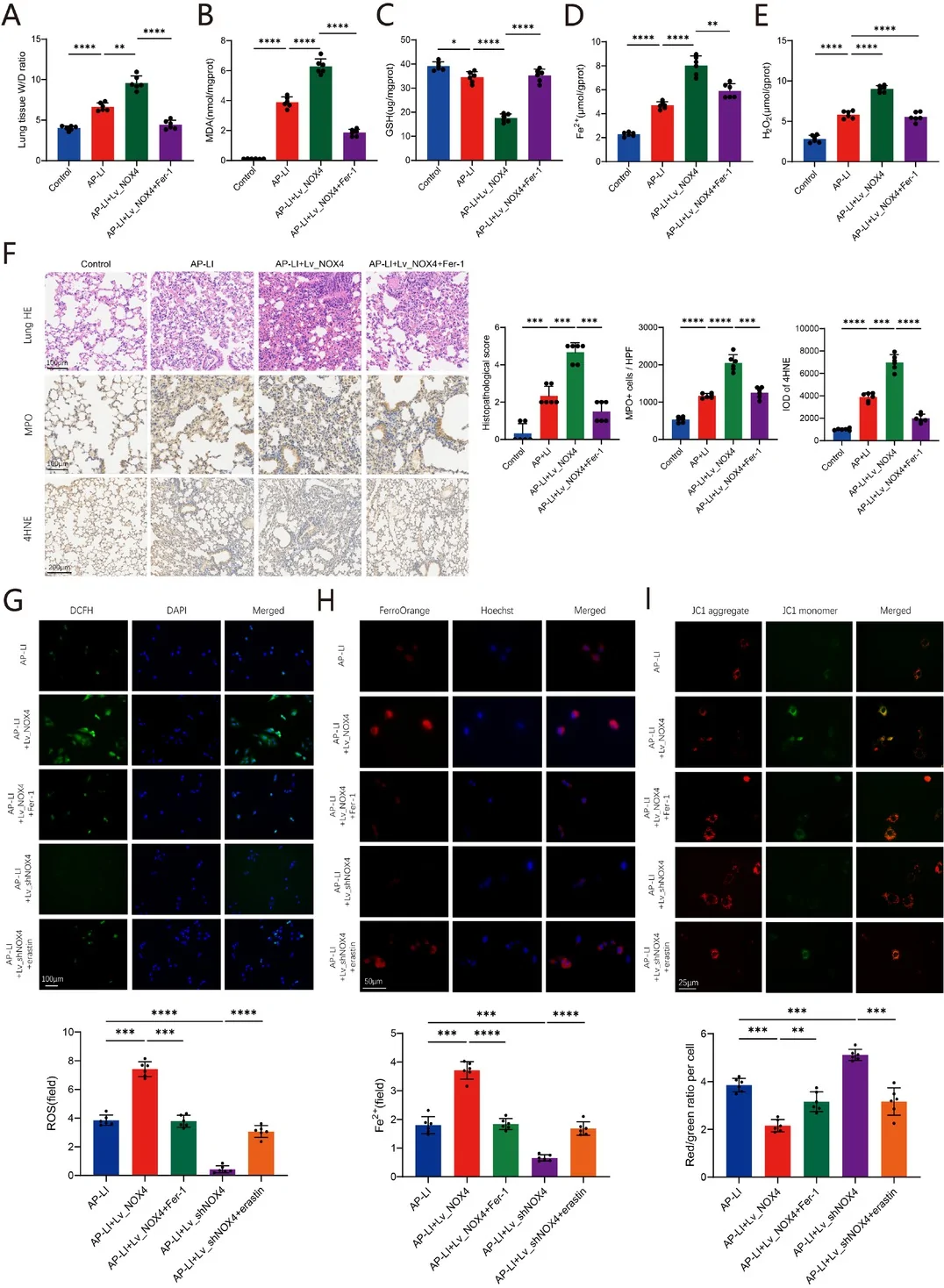
NOX4 exacerbates AT2-cell ferroptosis in AP-LI. (A-E) Lung tissue W/D ratio and MDA, GSH, Fe^2+^ and H_2_O_2_ levels in the control, AP-LI, AP-LI + Lv_NOX4, and AP-LI + Lv_NOX4 + Fer-1 groups. (F) Representative H&E and IHC staining images and quantitative analysis of lung tissues. Scale bars = 100 μm and 200 μm. (G) Representative images and quantitative analysis of ROS detected by DCFH/DAPI staining. Scale bar = 100 μm. (H) Representative images and quantitative analysis of Fe^2+^ detected by FerroOrange. Scale bar = 50 μm. (I) Representative images and quantitative analysis of the MMP visualized with JC-1 staining. Scale bar = 25 μm. The data are presented as the mean ± SD (A, B, D-F: *n* = 3; C: *n* = 6). Statistical significance was determined by one-way ANOVA followed by Tukey’s multiple comparisons test. ^*^P < 0.05, ^**^P < 0.01, ^***^P < 0.001, ^****^P < 0.0001. NOX4: NADPH oxidase 4; AT2: alveolar type II epithelial; AP-LI: acute pancreatitis-associated lung injury; W/D: wet-to-dry; MDA: malondialdehyde; GSH: glutathione; Lv_NOX4: NOX4 overexpression; Fer-1: ferrostatin-1; IHC: immunohistochemistry; ROS: reactive oxygen species; MMP: mitochondrial membrane potential.

### Plasma exosomal miR-25-3p post-transcriptionally represses NOX4 in AP-LI

Because miRNAs typically regulate gene expression by binding to 3′ UTR of target mRNAs and repressing translation without necessarily reducing transcript abundance,^[25]^ we next explored the downstream targets through which miR-25-3p may regulate ferroptosis. Potential target genes were predicted using miRDB. Among 919 predicted targets, 18 overlapped with ferroptosis-related genes in the FerrDb database (Figure 4A), and NOX4 had the highest prediction score (Figure 4B). To verify that miR-25-3p engages the RISC, AGO2-RIP was performed in AT2 cells using anti-AGO2 or IgG control antibodies, followed by RT-qPCR (Figure 4C). miR-25-3p enrichment in the AGO2 immunoprecipitate was significantly greater than that in the IgG control, confirming its association with RISC (Figure 4D). We then assessed NOX4 mRNA enrichment in AGO2 complexes after miR-25-3p mimic transfection. Compared with the NC mimic, the miR-25-3p mimic significantly increased NOX4 mRNA enrichment in AGO2-IP samples, indicating enhanced recruitment of NOX4 mRNA into RISC (Figure 4E). Dual-luciferase reporter assays further confirmed direct binding between miR-25-3p and the NOX4 3′ UTR (Figure 4F, G). *In vivo*, modulation of miR-25-3p did not significantly alter NOX4 mRNA levels but did significantly change NOX4 protein expression (Figure 4H, I). Specifically, miR-25-3p overexpression decreased NOX4 protein abundance, whereas miR-25-3p knockdown increased it. The same pattern was confirmed in primary AT2 cells by qPCR and Western blotting after transfection with a miR-25-3p mimic or inhibitor (Figure 4J, K). Together, these results show that miR-25-3p acts upstream of NOX4 and represses NOX4 expression predominantly at the post-transcriptional level.

### NOX4 promotes AT2-cell ferroptosis in AP-LI

NOX4 is a major source of ROS and oxidative stress is closely linked to ferroptosis, so we further investigated the role of NOX4 in AP-LI. NOX4 was overexpressed in mice by tail vein injection of lentiviral vectors carrying NOX4, and the efficiency of overexpression was confirmed by qPCR and Western blotting (Supplementary Figure S7A, B). Mice in the intervention group were treated with Fer-1 before AP-LI induction. H&E staining, histopathological scoring, and immunohistochemistry showed that NOX4 overexpression aggravated lung injury, whereas Fer-1 markedly attenuated these effects (Figure 5A-F). To further define the role of NOX4 *in vitro*, lentiviral vectors expressing NOX4 (Lv_NOX4) or shRNA targeting NOX4 (Lv_shNOX4) were introduced into primary AT2 cells, and efficient overexpression or knockdown was confirmed by Western blotting and qPCR (Supplementary Figure S7C-E). NOX4 knockdown reduced intracellular ROS and Fe^2+^ levels and increased MMP, whereas these effects were reversed by erastin. In contrast, NOX4 overexpression increased intracellular ROS and Fe^2+^ levels and decreased MMP, and these changes were attenuated by Fer-1 (Figure 5G-I). Collectively, these findings indicate that NOX4 is a downstream effector of miR-25-3p and a direct promoter of AT2-cell ferroptosis in AP-LI.

### NOX4 promotes ferroptosis through NCOA4-mediated ferritinophagy in AP-LI

We next investigated the mechanism linking NOX4 to ferroptosis. In AT2 cells from AP-LI mice, NOX4 overexpression increased intracellular Fe^2+^ levels and altered ferritin content (Figure 6A, B), suggesting that ferritin turnover may be involved. We therefore examined NCOA4-mediated ferritinophagy as a potential downstream pathway. Autophagic flux was assessed by fluorescence analysis in four groups of cells (Figure 6C). Compared with the control group, ferritinophagy was significantly increased in the AP-LI group. NOX4 overexpression further enhanced ferritinophagy, whereas NOX4 knockdown reduced it. In parallel, total protein was extracted from AT2 cells in the control, AP-LI, AP-LI + Lv_NOX4, and AP-LI + Lv_shNOX4 groups, and Western blotting was used to assess NOX4 and ferritinophagy-related proteins (Figure 6D). To further verify this mechanism, primary AT2 cells from AP-LI mice were subjected to NOX4 overexpression together with NCOA4 knockdown (Lv_shNCOA4). NCOA4 knockdown, as well as treatment with the autophagy inhibitor 3-MA, effectively attenuated the reduction in ferritin levels induced by NOX4 overexpression (Figure 6E, F). These data indicate that NCOA4-mediated ferritinophagy functions downstream of NOX4 and contributes to NOX4-driven ferroptotic susceptibility.

**Figure 6:**
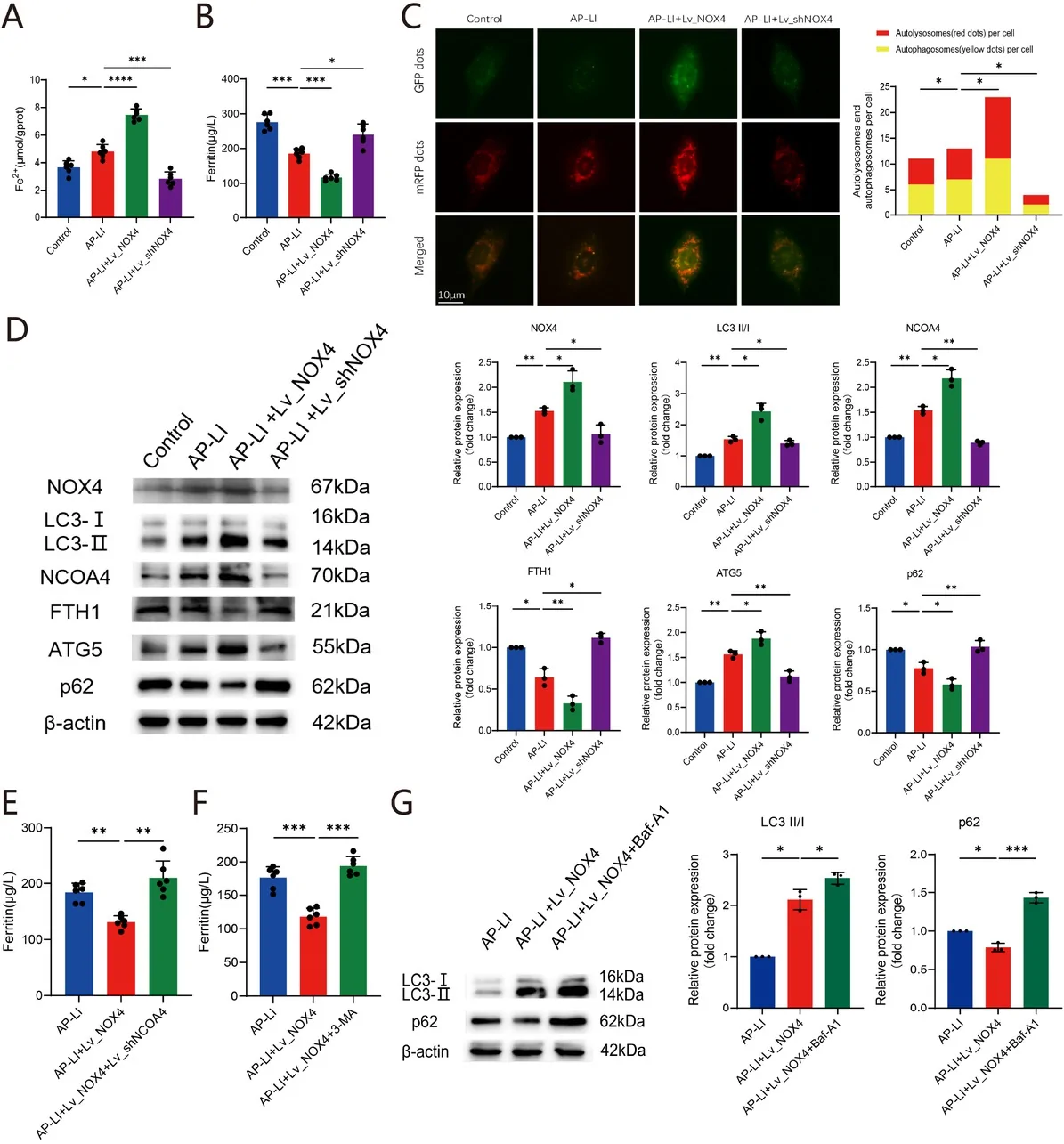
NOX4 regulates ferroptosis in AP-LI through the NCOA4-mediated ferritinophagy pathway. (A, B) Fe^2+^ and ferritin levels in AT2 control, AP-LI, AP-LI + Lv_NOX4 and AP-LI + Lv_shNOX4 cells. (C) Representative fluorescence images of autophagic flux in mRFP-GFP-LC3-tagged AT2 cells subjected to control, AP-LI, AP-LI + Lv_NOX4 and AP-LI + Lv_shNOX4 treatments. The number of autophagic dots and the ratio of red dots (autolysosomes) to yellow dots (autophagosomes) per cell were calculated. Bar = 10 μm. (D) Representative Western blot images and quantitative analysis of NOX4, LC3-II/I, NCOA4, FTH1, ATG5, and p62 protein levels in AT2 cells from the control, AP-LI, AP-LI + Lv_NOX4 and AP-LI + Lv_shNOX4 groups. β-Actin was used as the protein loading control. (E) Ferritin levels in AP-LI, AP-LI + Lv_NOX4, and AP-LI + Lv_NOX4 + Lv_shNCOA4 cells. (F) Ferritin levels in AP-LI, AP-LI + Lv_NOX4, and AP-LI + Lv_NOX4 + 3-MA cells. (G) Representative Western blot images and quantitative analysis of LC3-II/I, and p62 protein levels in AT2 cells from the AP-LI, AP-LI + Lv_NOX4 and AP-LI + Lv_NOX4 + Baf-A1 groups. β-Actin was used as the protein loading control. The data are presented as the mean ± SD (A-C, E, F: *n* = 6, D, G: *n* = 3). Statistical significance was determined by one-way ANOVA followed by Tukey’s multiple comparisons test. ^*^P < 0.05, ^**^P < 0.01, ^***^P < 0.001, ^****^P < 0.0001, ns not significant. NOX4: NADPH oxidase 4; AT2: alveolar type II epithelial; AP-LI: acute pancreatitis-associated lung injury; NCOA4: nuclear receptor coactivator 4; Lv_shNOX4: NOX4 knockdown; LC3: microtubule-associated protein 1 light chain 3; FTH1: ferritin Heavy Chain 1; ATG5: autophagy related 5; 3-MA: 3-methyladenine; Baf-A1: bafilomycin A1.

To quantitatively distinguish enhanced autophagic flux from impaired lysosomal degradation, we performed lysosomal inhibition experiments using bafilomycin A1 (Baf-A1). Primary AT2 cells were treated with Baf-A1 under conditions of NOX4 overexpression, and LC3 and p62 levels were assessed by Western blotting. Compared with the corresponding conditions without Baf-A1, Baf-A1 treatment caused marked accumulation of LC3-II and p62, indicating preserved lysosomal degradative capacity and active autophagic flux (Figure 6G). These findings provide quantitative evidence that NOX4 promotes NCOA4-mediated ferritinophagy by enhancing autophagic flux rather than by blocking lysosomal clearance.

### NOX4 activates HIF-1α-dependent NCOA4 transcription to enhance ferritinophagy

To further define the downstream signaling cascade linking NOX4 to NCOA4-mediated ferritinophagy, we examined HIF-1α activation in primary AT2 cells. NOX4 overexpression markedly increased HIF-1α expression and enhanced NCOA4/FTH1-associated ferritinophagy, especially under AP-LI conditions; these effects were reversed by N-acetyl-L-cysteine (NAC), a ROS scavenger (Figure 7A). Immunofluorescence analysis showed that ROS accumulation and HIF-1α expression followed the same pattern as observed by Western blotting (Figure 7C, D). Moreover, HIF-1α overexpression significantly enhanced NCOA4/FTH1-mediated ferritinophagy in NOX4-overexpressing AT2 cells, whereas pharmacological inhibition of HIF-1α with YC-1 attenuated this effect (Figure 7B). To characterize the regulatory relationship between HIF-1α and NCOA4, we analyzed the correlation between their expression levels and predicted putative HIF-1α binding sites within the NCOA4 promoter. Site 7 showed the strongest enrichment, and the region spanning positions 1519 to 1523 was therefore selected for further ChIP analysis (Figure 7E-H). Dual-luciferase reporter assays showed that HIF-1α overexpression significantly increased the transcriptional activity of the wild-type NCOA4 promoter, whereas mutation of the HIF-1α binding site abolished this effect (Figure 7I, J). These results demonstrate that HIF-1α directly binds to the hypoxia response element (HRE) in the NCOA4 promoter and positively regulates NCOA4 transcription. Collectively, these data indicate that NOX4-derived ROS promotes ferritinophagy by activating HIF-1α-dependent transcription of NCOA4.

**Figure 7:**
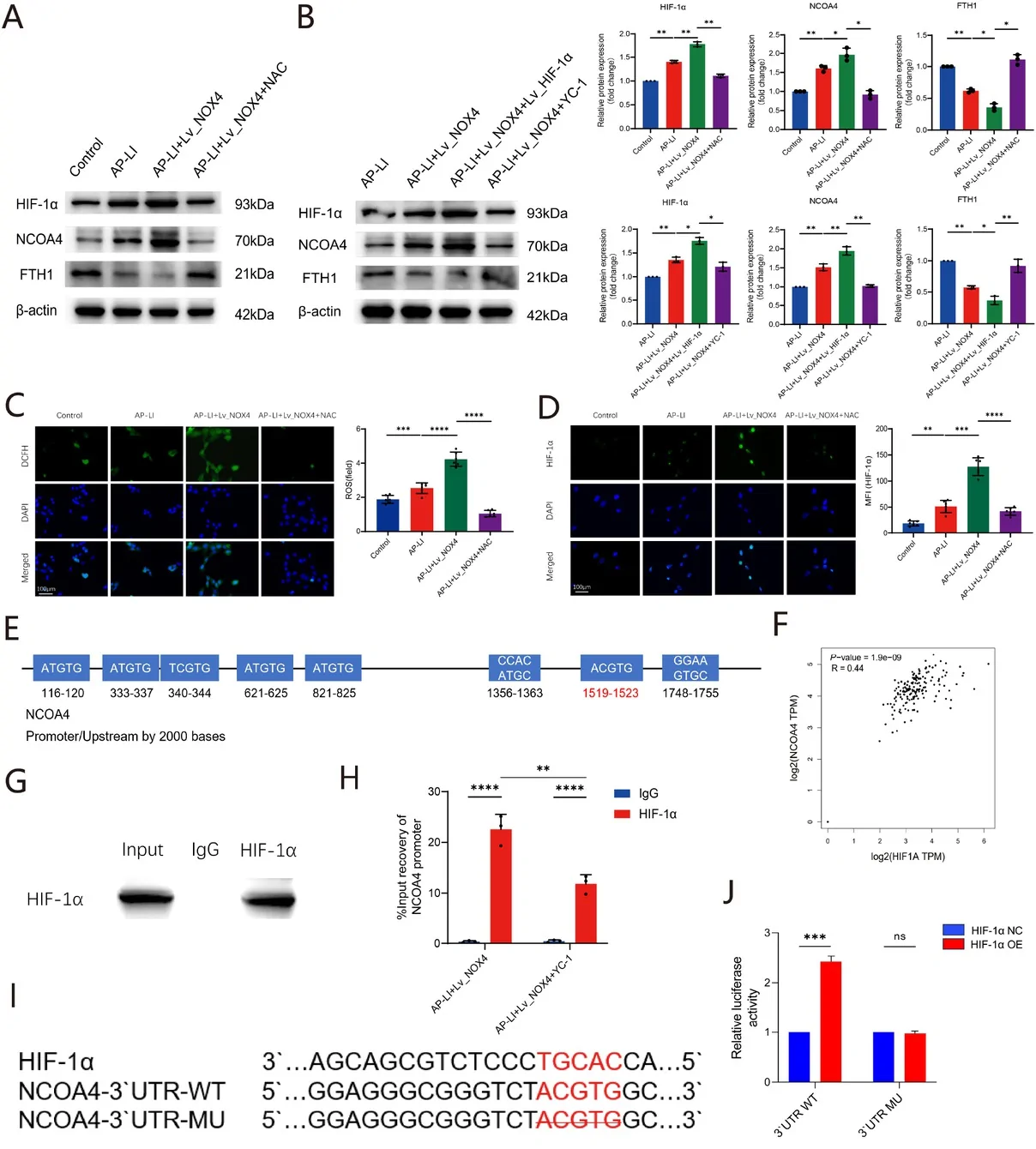
NOX4 enhances NCOA4/FTH1-mediated ferritinophagy by upregulating the transcriptional activity of HIF-1α. (A) Representative Western blot images and quantitative analysis of HIF-1α, NCOA4 and FTH1 in AT2 cells treated with control, AP-LI, AP-LI + Lv_NOX4 and AP-LI + Lv_NOX4 + NAC. β-actin was used as the protein loading control. (B) Representative Western blot images and quantitative analysis of HIF-1α, NCOA4 and FTH1 in AT2 cells treated with AP-LI, AP-LI + Lv_NOX4, AP-LI + Lv_NOX4 + Lv_HIF-1α and AP-LI + Lv_NOX4 + YC-1. β-actin was used as the protein loading control. (C) Representative images and quantitative analysis of ROS in AT2 cells with DCFH staining. (D) Representative images and quantitative analysis of HIF-1α expression in AT2 cells. (E) Interaction region predicted between HIF-1α and NCOA4 promoter. (F) Correlation analysis of HIF-1α and NCOA4 expression using GEPIA. (G) ChIP was performed in AT2 cells against IgG or HIF-1α. (H) % input enrichment of the NCOA4 promoter with IgG and HIF-1α in AT2 cells treated with AP-LI + Lv_NOX4 and AP-LI + Lv_NOX4 + YC-1. The data are presented as the mean ± SD (A, B, H: *n* = 3, C, D: *n* = 6). (I, J) Luciferase activity assays showing the effect of HIF-1α on wild-type and mutant NCOA4 promoter reporters. The data are presented as the mean ± SD (*n* = 3). Differences were analyzed using a two-tailed unpaired Student’s t test. Statistical significance was determined by one-way ANOVA followed by Tukey’s multiple comparisons test. ^*^P < 0.05, ^**^P < 0.01, ^***^P < 0.001, ^****^P < 0.0001, ns not significant. NOX4: NADPH oxidase 4; AT2: alveolar type II epithelial; AP-LI: acute pancreatitis-associated lung injury; NCOA4: nuclear receptor coactivator 4; ChIP: chromatin immunoprecipitation; ROS: reactive oxygen species; HIF-1α: hypoxia inducible factor 1 subunit alpha; Lv_NOX4: NOX4 overexpression; FTH1: ferritin Heavy Chain 1; YC-1: lificiguat.

### The miR-25-3p mimic counteracts NOX4-driven ferroptosis in vivo and in vitro

To further validate the functional relationship between miR-25-3p and NOX4 in ferroptosis regulation, rescue experiments were performed *in vivo* and *in vitro*. Lung tissues and primary AT2 cells from AP-LI mice were assigned to four groups: (1) AP-LI control, (2) AP-LI + lentiviral Lv_NOX4, (3) AP-LI + miR-25-3p mimic, and (4) AP-LI + Lv_NOX4 + miR-25-3p mimic. *In vitro*, Western blotting showed that NOX4 overexpression significantly decreased glutathione peroxidase 4 (GPX4) and ferritin heavy chain 1 (FTH1) protein levels while increasing acyl-CoA synthetase long chain family member 4 (ACSL4) and NCOA4 expression; these changes were effectively reversed by cotransfection with the miR-25-3p mimic (Figure 8A). Consistently, DCFH-DA fluorescence assays revealed that NOX4 overexpression increased intracellular ROS levels in AT2 cells, whereas miR-25-3p mimic treatment markedly attenuated this increase (Figure 8B). *In vivo*, Western blotting and DHE fluorescence imaging of lung tissue yielded concordant results, showing parallel changes in ferroptosis-related protein expression and ROS accumulation (Figure 8C, D). Transmission electron microscopy further demonstrated classic ferroptotic ultrastructural changes, including mitochondrial shrinkage, chromatin condensation, and disruption of the inner mitochondrial membrane, in AT2 cells from the Lv_NOX4 group; these abnormalities were substantially alleviated by miR-25-3p mimic administration (Figure 8E). In addition, NOX4 knockdown in AP-LI-derived AT2 cells reduced the incremental protective effect of the miR-25-3p mimic on ferroptosis and ferritinophagy (Supplementary Figure S8A), further supporting NOX4 as a key downstream effector of miR-25-3p. Together, these findings show that miR-25-3p mitigates AP-LI largely by antagonizing NOX4-driven ferroptosis.

**Figure 8:**
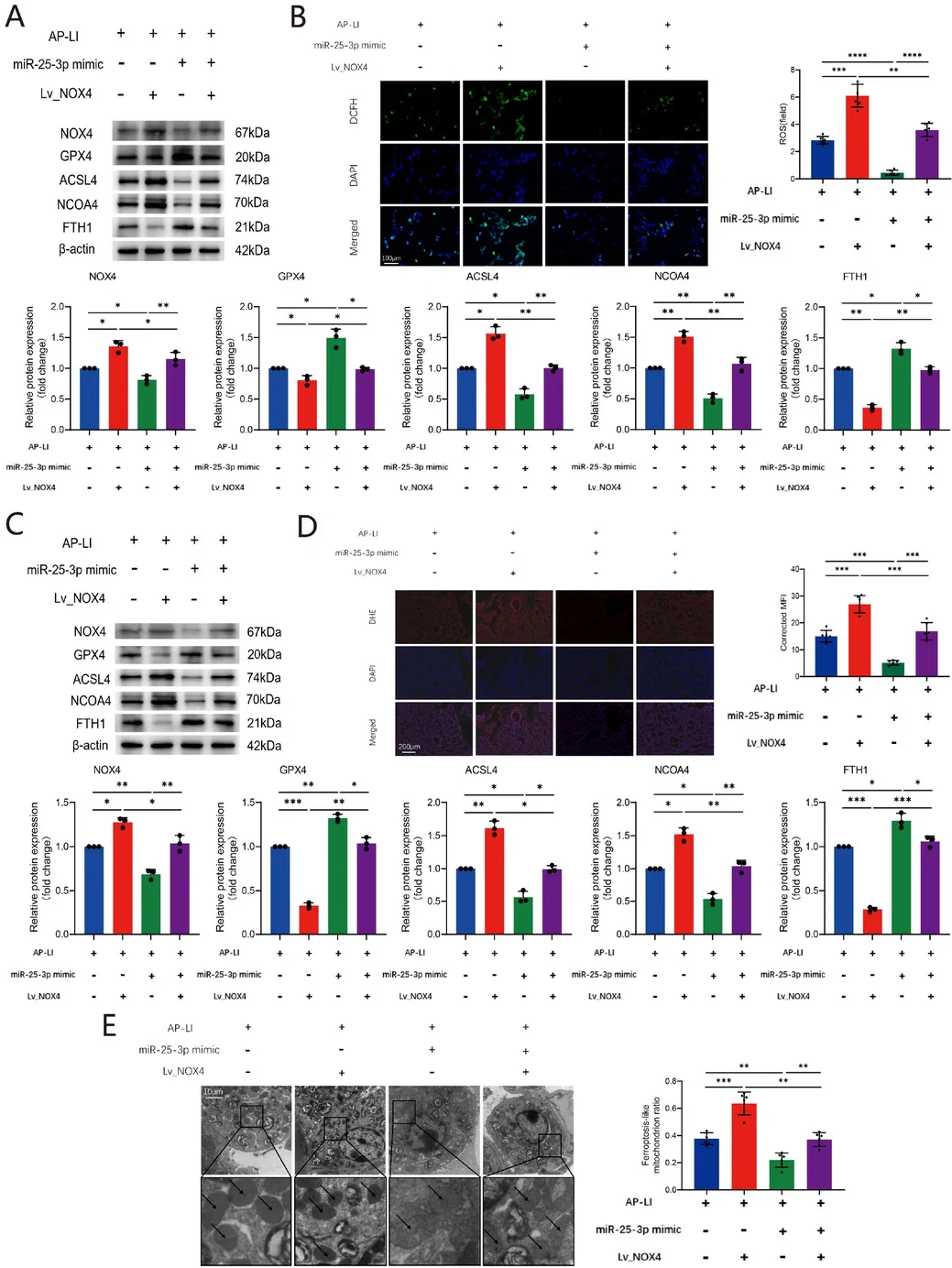
The miR-25-3p mimic attenuates NOX4-mediated ferroptosis in vivo and in vitro. (A) Representative Western blot images and quantitative analysis of NOX4, GPX4, ACSL4, NCOA4 and FTH1 expression in AT2 cells that were treated with AP-LI, AP-LI + Lv_NOX4, AP-LI + miR-25-3p mimic or AP-LI + Lv_NOX4 + miR-25-3p mimic. β-actin was used as the protein loading control. (B) Representative images and quantitative analysis of ROS in AT2 cells with DCFH staining. Scale bar = 100 μm. (C) Representative Western blot images and quantitative analysis of NOX4, GPX4, ACSL4, NCOA4 and FTH1 expression in lung tissues that were treated with AP-LI, AP-LI + Lv_NOX4, AP-LI + miR-25-3p mimic or AP-LI + Lv_NOX4 + miR-25-3p mimic. β-actin was used as the protein loading control. (D) Representative images and quantitative analysis of ROS in lung tissues with DHE staining. Scale bar = 200 μm. (E) Representative TEM images and quantitative analysis of AT2 cells in lung tissues showing mitochondrial morphology. Scale bar = 10 μm. The data are presented as the mean ± SD (A, C: *n* = 3; B, D, E: *n* = 6). Statistical significance was determined by one-way ANOVA followed by Tukey’s multiple comparisons test. ^*^P < 0.05, ^**^P < 0.01, ^***^P < 0.001, ^****^P < 0.0001, ns not significant. NOX4: NADPH oxidase 4; NCOA4: nuclear receptor coactivator 4; GPX4: glutathione peroxidase 4; ACSL4: acyl-CoA synthetase long chain family member 4; FTH1: ferritin Heavy Chain 1; AT2: alveolar type II epithelial; AP-LI: acute pancreatitis-associated lung injury; Lv_NOX4: NOX4 overexpression; ROS: reactive oxygen species; DHE: dihydroethidium; Lv_shNOX4: NOX4 knockdown; TEM: transmission electron microscopy.

### Plasma exosomal miR-25-3p is downregulated in AP patients and inversely correlates with disease severity

To assess the clinical relevance of plasma exosomal miR-25-3p in AP, peripheral blood samples were collected from three cohorts: 15 patients with mild-to-moderate AP, 15 patients with severe AP complicated by acute lung injury, and 15 healthy controls (Table S5). Plasma exosomes were isolated and purified by ultracentrifugation followed by size-exclusion chromatography. Transmission electron

microscopy and NanoFCM confirmed the characteristic cup-shaped morphology and size distribution (30-150 nm) of exosomes in all groups. However, comparative analyses revealed subtle but consistent differences in particle size distribution and reduced expression of the canonical exosomal markers CD63, CD81, and TSG101 in exosomes from AP patients relative to those from healthy controls (Figure 9A-D). Real-time PCR showed that plasma exosomal miR-25-3p levels were significantly decreased in both the mild-to-moderate AP and severe AP groups compared with the healthy control group (Figure 9E). Pearson correlation analysis further showed that plasma exosomal miR-25-3p expression was negatively correlated with serum amylase and lipase levels (Figure 9F, G) and positively correlated with the pulmonary oxygenation index (Figure 9H), suggesting that lower circulating exosomal miR-25-3p levels are associated with greater AP severity and more severe lung injury.

**Figure 9:**
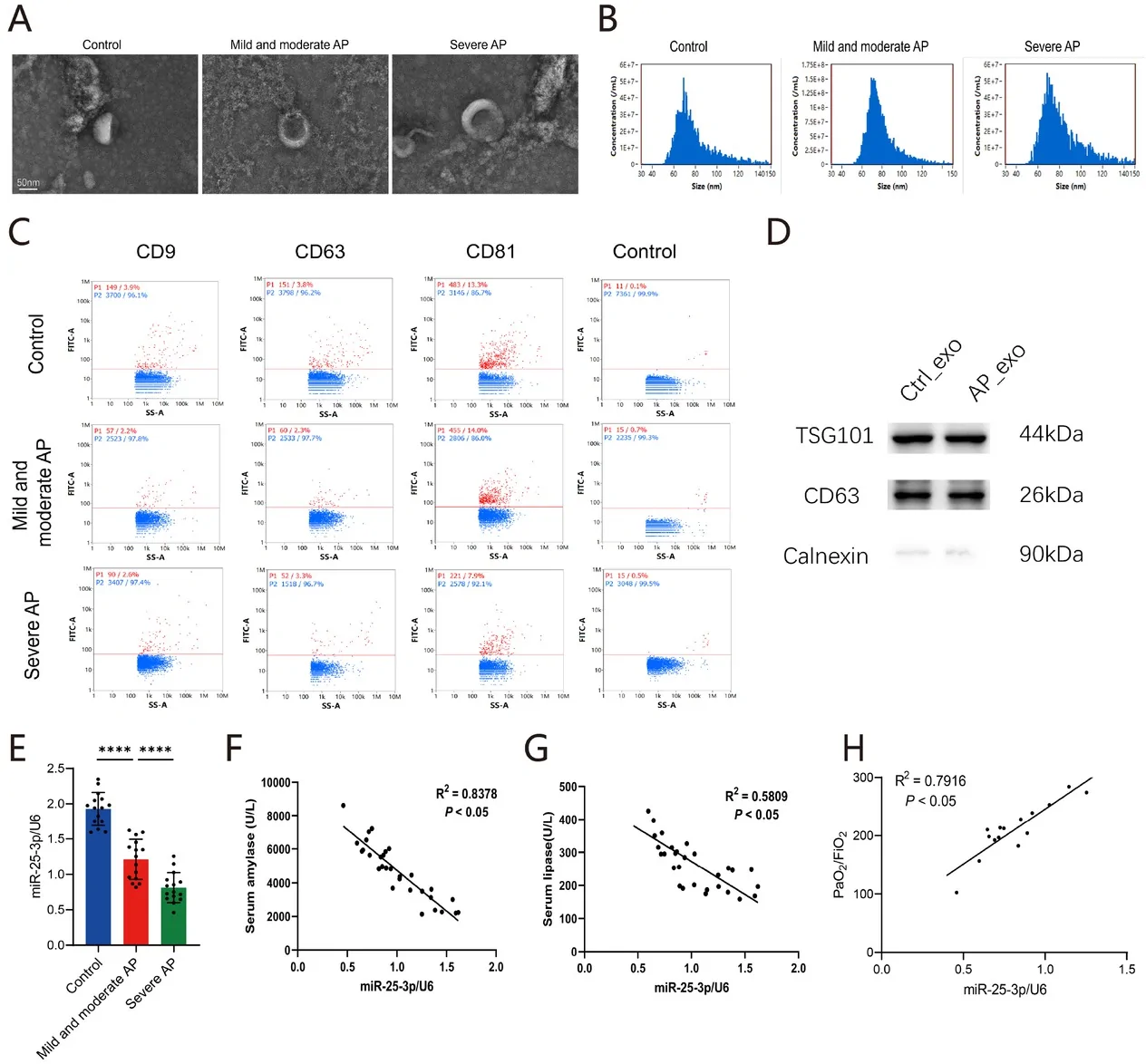
Plasma exosomal miR-25-3p expression is downregulated in AP patients and inversely correlates with disease severity. (A) Representative TEM images of plasma exosomes. Scale bar = 50 nm. (B) Particle size and concentration of plasma exosomes. (C) Surface marker analysis (CD9, CD63, and CD81) of plasma exosomes. (D) Protein expression levels of exosomal markers (including TSG101, CD63 and calnexin). (E) miR-25-3p level in plasma exosomes of healthy controls, mild-to-moderate AP patients, severe AP patients. (F, G) Correlation analysis between plasma exosome miR-25-3p expression and serum amylase levels, serum lipase levels in 30 AP patients. (H) Correlation analysis between plasma exosome miR-25-3p expression and pulmonary oxygenation index in 15 severe AP patients. Data are presented as mean ± SD, group comparison: *n* = 15; amylase/Lipase correlation: *n* = 30; oxygenation index correlation: *n* = 15. Statistical significance was determined by one-way ANOVA followed by Tukey’s multiple comparisons test. Correlation analysis was performed using Pearson’s correlation coefficient. ^****^P < 0.0001. AP: acute pancreatitis; TEM: transmission electron microscopy.

## Discussion

Inflammation and oxidative stress are central to the pathogenesis of AP and its pulmonary complications, but the mechanistic links between pancreatic injury and cell-type-specific lung damage remain incompletely understood.^[26]^ AP-LI is a prototypical form of remote organ injury in which the primary pancreatic insult rapidly propagates systemic danger signals that disrupt the alveolar-capillary unit. Although cytokines and damage-associated molecular patterns have long been implicated in this process, the molecular pathway that connects circulating signals to a defined death program in a specific pulmonary cell population has remained unclear. Our data support a model in which plasma exosomal miR-25-3p acts as an upstream regulator of alveolar epithelial stress responses by repressing NOX4, thereby limiting downstream ferritinophagy-dependent ferroptosis in AT2 cells.

Exosomes are key mediators of intercellular communication and contribute to cellular homeostasis, immune regulation, and disease progression across a broad spectrum of physiological and pathological contexts.^[27, 28, 29]^ By transferring miRNAs, exosomes can alter post-transcriptional programs in recipient cells through mRNA degradation or translational repression.^[30, 31, 32]^ In AP, exosome-associated miRNAs are increasingly recognized as mediators of interorgan communication and complication development. Injured pancreatic acinar cells appear to be an important source of pathogenic exosomes and may drive pancreas-to-remote-organ crosstalk.^[33,34]^ Previous studies have shown that exosomal miRNAs isolated from plasma or ascites can influence AP progression and organ dysfunction by reprogramming inflammatory and stress-response pathways.^[35,36]^ Within this context, miR-25-3p is particularly notable because it has shown cytoprotective and antioxidative effects in multiple injury models, supporting its biological relevance in AP-LI.^[37, 38, 39]^

Among the regulated cell death programs implicated in AP, ferroptosis has attracted particular attention because it directly links oxidative stress, iron dysregulation, and organ injury. Ferroptosis is an iron-dependent oxidative cell death process characterized by excess iron burden, lipid peroxidation, and ROS accumulation.^[40,41]^ In AP, excessive ROS production and disordered iron metabolism amplify inflammatory injury and provide a biological basis for remote organ damage driven by ferroptosis.^[42, 43, 44]^ Experimental inhibition of ferroptosis attenuates oxidative stress, dampens systemic inflammation, and alleviates multiorgan injury, including lung and kidney injury.^[45,46]^ In acute lung injury models, ferroptosis has likewise been implicated in alveolar epithelial damage and inflammatory amplification, further supporting its relevance to AP-LI.^[47,48]^

NOX4 is a major enzymatic source of ROS and has been implicated in both pancreatic injury and remote organ dysfunction during AP.^[49,50]^ In the lung, increased NOX4 activity has been linked to alveolar inflammation, edema, and epithelial injury.^[51, 52, 53]^ NCOA4, in contrast, functions as a selective ferritinophagy receptor that mobilizes intracellular iron and thereby links autophagy to ferroptotic susceptibility.^[54,55]^ The present study clarifies the directional relationship among these molecules. Specifically, miR-25-3p functions upstream as a negative regulator of NOX4; NOX4 then acts downstream to promote ROS accumulation; and NCOA4-mediated ferritinophagy functions further downstream to increase iron availability and ferroptotic vulnerability in AT2 cells. Our data additionally suggest that HIF-1α serves as a transcriptional intermediate linking NOX4-derived ROS to NCOA4 upregulation. This sequence helps define a more coherent pathway framework for AP-LI, rather than viewing oxidative stress, ferritinophagy, and ferroptosis as parallel but disconnected events.

Several limitations and translational challenges should also be acknowledged. First, the mechanistic evidence in this study is derived predominantly from murine models and *in vitro* experiments, whereas the clinical cohort is relatively small. Validation in larger, well-phenotyped patient populations will therefore be necessary. Second, although our data support an important role for the miR-25-3p/NOX4/NCOA4/ferroptosis axis in AT2 cells, its relevance in other pulmonary cell populations, including alveolar macrophages, endothelial cells, and AT1 cells, remains unclear. Third, administration of miR-25-3p-enriched engineered exosomes only partially recapitulates endogenous vesicle-mediated communication and may not fully reflect the kinetics, tissue origin, cargo heterogeneity, or context dependence of circulating exosomes during AP-LI. These issues should be addressed in future studies to better define the cellular specificity and temporal hierarchy of this signaling axis.

The translational applicability of exosome-based miR-25-3p delivery also remains uncertain. Major barriers include cargo-loading efficiency, batch-to-batch reproducibility, *in vivo* pharmacokinetics, tissue biodistribution, potential immunogenicity under conditions of systemic inflammation, and off-target effects related to the pleiotropic nature of miR-25-3p and the broader physiological functions of NOX4. In addition, scalable manufacturing, purification standardization, and long-term storage stability of engineered exosomes remain important practical challenges. These issues will need to be resolved before therapeutic development can be considered realistic.

## Conclusion

In conclusion, our study supports an integrated mechanistic model in which plasma exosomal miR-25-3p restrains NOX4-driven oxidative stress, thereby reducing HIF-1α-dependent NCOA4 transcription, ferritinophagy, and AT2-cell ferroptosis in AP-associated remote lung injury (Figure 10). By defining this upstream-to-downstream regulatory sequence, our work provides a more coherent framework for understanding how exosome-mediated pancreas-lung communication promotes AP-LI. These findings support the biological relevance of the miR-25-3p/NOX4/NCOA4 axis and warrant further validation in clinically relevant settings.

**Figure 10:**
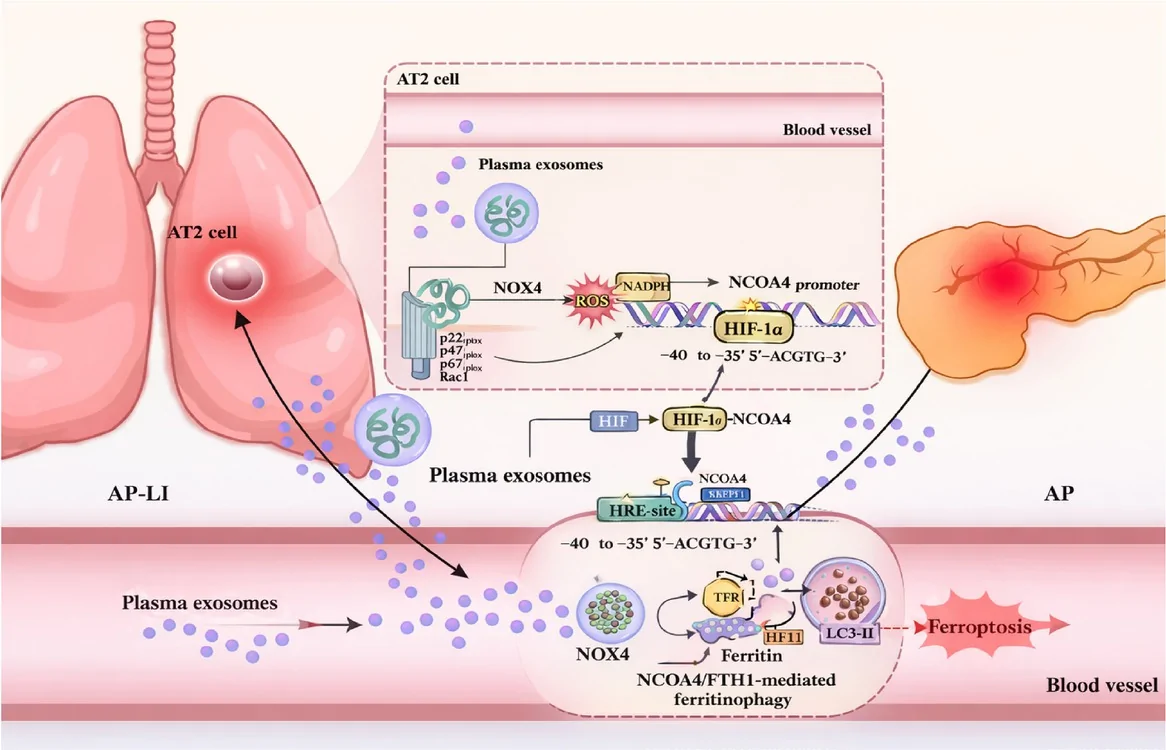
Schematic diagram of the mechanism through which plasma exosomal miR-25-3p regulates AP-LI via NOX4/NCOA4-mediated ferritinophagy in AT2 cells. AP-LI: acute pancreatitis-associated lung injury; AP: acute pancreatitis; NOX4: NADPH oxidase 4; NCOA4: nuclear receptor coactivator 4; FTH1: ferritin Heavy Chain 1; AT2: alveolar type II epithelial; HIF-1α: hypoxia inducible factor 1 subunit alpha; TFR: Transferrin receptor. This figure was created with Adobe Photoshop 2024 (Adobe Inc., San Jose, CA, USA).

## Supplementary Information

Supplementary materials are only available at the journal’s official site at: www.intern-med.com.

## Supplementary Material

Supplementary Material Details

## References

[1] Iannuzzi JP, King JA, Leong JH (2022). Global incidence of acute pancreatitis is increasing over time: a systematic review and meta-analysis. Gastroenterology.

[2] Luo Y, Li Z, Ge P (2021). Comprehensive mechanism, novel markers and multidisciplinary treatment of severe acute pancreatitis-associated cardiac injury–a narrative review. J Inflamm Res.

[3] Zhou J, Zhou P, Zhang Y, Wang G, Fan Z (2021). Signal pathways and markers involved in acute lung injury induced by acute pancreatitis. Dis Markers.

[4] Dixon SJ, Lemberg KM, Lamprecht MR (2012). Ferroptosis: an iron-dependent form of nonapoptotic cell death. Cell.

[5] Gu X, Huang Z, Ying X (2024). Ferroptosis exacerbates hyperlipidemic acute pancreatitis by enhancing lipid peroxidation and modulating the immune microenvironment. Cell Death Discov.

[6] Yang H, Liu Y, Yao J (2024). Mesenchymal stem cells inhibit ferroptosis by activating the Nrf2 antioxidation pathway in severe acute pancreatitis-associated acute lung injury. Eur J Pharmacol.

[7] Palumbo S, Shin Y-J, Ahmad K (2017). Dysregulated Nox4 ubiquitination contributes to redox imbalance and age-related severity of acute lung injury. Am J Physiol Lung Cell Mol Physiol.

[8] Li ZM, Xu SY, Feng YZ (2021). The role of NOX4 in pulmonary diseases. J Cell Physiol.

[9] Wang J, Wu N, Peng M (2023). Ferritinophagy: research advance and clinical significance in cancers. Cell Death Discov.

[10] Sciarretta S, Zhai P, Shao D (2013). Activation of NADPH oxidase 4 in the endoplasmic reticulum promotes cardiomyocyte autophagy and survival during energy stress through the protein kinase RNA-activated-like endoplasmic reticulum kinase/eukaryotic initiation factor 2α/activating transcription factor 4 pathway. Circ Res.

[11] Jia C, Lin HJ, Cui F (2025). [Research progress on NCOA4-mediated ferritinophagy and related diseases]. Acta Physiol Sin.

[12] Ou H, Lin J, Ji L (2024). Ferritinophagy mediated by the AMPK/ULK1 pathway is involved in ferroptosis subsequent to ventilator-induced lung injury. Respir Res.

[13] Liu G, Zhang J, Chen H (2014). Effects and mechanisms of alveolar type II epithelial cell apoptosis in severe pancreatitis-induced acute lung injury. Exp Ther Med.

[14] Yin X, Zhu G, Wang Q, Fu YD, Wang J, Xu B (2021). Ferroptosis, a new insight into acute lung injury. Front Pharmacol.

[15] Zhang Y, Xie J (2024). Ferroptosis-related exosomal non-coding RNAs: promising targets in pathogenesis and treatment of non-malignant diseases. Front Cell Dev Biol.

[16] Xiang H, Tao X, Xia S (2017). Targeting microRNA function in acute pancreatitis. Front Physiol.

[17] Qu Y, Ding Y, Lu J (2023). Identification of key microRNAs in exosomes derived from patients with the severe acute pancreatitis. Asian J Surg.

[18] Varga ZV, Kupai K, Szűcs G (2013). microRNA-25-dependent up-regulation of NADPH oxidase 4 (NOX4) mediates hypercholesterolemia-induced oxidative/nitrative stress and subsequent dysfunction in the heart. J Mol Cell Cardiol.

[19] Jiang X, Guo S, Xu M (2022). TFAP2C-Mediated lncRNA PCAT1 Inhibits Ferroptosis in Docetaxel-Resistant Prostate Cancer Through c-Myc/miR-25-3p/SLC7A11 Signaling. Front Oncol.

[20] Bayat M, Sadri Nahand J (2024). Exosomal miRNAs: the tumor’s Trojan horse in selective metastasis. Mol Cancer.

[21] Niederau C, Ferrell LD, Grendell JH (1985). Caerulein-induced acute necrotizing pancreatitis in mice; protective effects of proglumide benzotript, and secretin. Gastroenterology.

[22] Yang X, Yao L, Fu X (2020). Experimental acute pancreatitis models: history, current status, and role in translational research. Front Physiol.

[23] Szarka RJ, Wang N, Gordon L, Nation PN, Smith RH (1997). A murine model of pulmonary damage induced by lipopolysaccharide via intranasal instillation. J Immunol Meth.

[24] Kusske AM, Rongione AJ, Ashley SW, McFadden DW, Reber HA (1996). Interleukin-10 prevents death in lethal necrotizing pancreatitis in mice. Surgery.

[25] Wei S, Julie Li Y-S, Huang H-D, Shyy JY, Shu C (2010). microRNA: a master regulator of cellular processes for bioengineering systems. Annu Rev Biomed Eng.

[26] Ge P, Luo Y, Okoye CS (2020). Intestinal barrier damage, systemic inflammatory response syndrome, and acute lung injury: a troublesome trio for acute pancreatitis. Biomed Pharmacother.

[27] Simpson RJ, Lim JW, Moritz RL, Mathivanan S (2009). Exosomes: proteomic insights and diagnostic potential. Expert Rev Proteom.

[28] Isaac R, Reis FCG, Ying W, Olefsky JM (2021). Exosomes as mediators of intercellular crosstalk in metabolism. Cell Metab.

[29] Yuan Y, Han X, Zhao X (2024). Circulating exosome long non-coding RNAs are associated with atrial structural remodeling by increasing systemic inflammation in atrial fibrillation patients. J Transl Intern Med.

[30] Bai S, Su X, Kong D (2025). Selective HDAC8 inhibition by PCI-34051 attenuates inflammation and airway remodeling in asthma via miR-381-3p-TGFβ3 axis. J Transl Intern Med.

[31] Manna I, De Benedittis S, Quattrone A, Maisano D, Iaccino E, Quattrone A (2020). Exosomal miRNAs as potential diagnostic biomarkers in Alzheimer’s disease. Pharmaceuticals.

[32] Valadi H, Ekström K, Bossios A, Sjöstrand M, Lee JJ, Lötvall JO (2007). Exosome-mediated transfer of mRNAs and microRNAs is a novel mechanism of genetic exchange between cells. Nat Cell Biol.

[33] Jiménez-Alesanco A, Marcuello M, Pastor-Jiménez M (2019). Acute pancreatitis promotes the generation of two different exosome populations. Sci Rep.

[34] Jia YC, Ding YX, Mei WT (2021). Extracellular vesicles and pancreatitis: mechanisms, status and perspectives. Int J Biol Sci.

[35] Yang Q, Luo Y, Lan B (2022). Fighting fire with fire: exosomes and acute pancreatitis-associated acute lung injury. Bioengineering.

[36] Xu Y, Sun Y, Yin R (2022). Differential expression of plasma exosomal microRNA in severe acute pancreatitis. Front Pharmacol.

[37] Peng Y, Zhao JL, Peng ZY, Xu WF, Yu GL (2020). Exosomal miR-25-3p from mesenchymal stem cells alleviates myocardial infarction by targeting pro-apoptotic proteins and EZH2. Cell Death Dis.

[38] Li H, Lin W, Zhang G (2023). BMSC-exosomes miR-25-3p regulates the p53 signaling pathway through pten to inhibit cell apoptosis and ameliorate liver Ischemia‒reperfusion injury. Stem Cell Rev Rep.

[39] Luo XY, Ying JH, Wang QS (2022). miR-25-3p ameliorates SAE by targeting the TLR4/NLRP3 axis. Metab Brain Dis.

[40] Chen X, Yu C, Kang R, Kroemer G, Tang D (2021). Cellular degradation systems in ferroptosis. Cell Death Differ.

[41] Sun Y, Chen P, Zhai B (2020). The emerging role of ferroptosis in inflammation. Biomed Pharmacother.

[42] Pădureanu V, Florescu D, Pădureanu R, Ghenea A, Gheonea D, Oancea C (2022). Role of antioxidants and oxidative stress in the evolution of acute pancreatitis (Review). Exp Ther Med.

[43] Esrefoglu M (2012). Experimental and clinical evidence of antioxidant therapy in acute pancreatitis. World J Gastroenterol.

[44] Yu JH (2014). Oxidative stress and inflammatory signaling in cerulein pancreatitis. World J Gastroenterol.

[45] Li H, Lin Y, Zhang L, Zhao J, Li P (2022). Ferroptosis and its emerging roles in acute pancreatitis. Chin Med J.

[46] Liu X, Wang L, Xing Q (2021). Sevoflurane inhibits ferroptosis: a new mechanism to explain its protective role against lipopolysaccharide-induced acute lung injury. Life Sci.

[47] Zhan J, Chen J, Deng L, Lu Y, Luo L (2024). Exploring the ferroptosis-related gene lipocalin 2 as a potential biomarker for sepsis-induced acute respiratory distress syndrome based on machine learning. Biochim Biophys Acta BBA Mol Basis Dis.

[48] Qiu B, Zandkarimi F, Saqi A (2024). Fatal COVID-19 pulmonary disease involves ferroptosis. Nat Commun.

[49] Sirokmány G, Donkó Á, Geiszt M (2016). Nox/duox family of NADPH oxidases: lessons from knockout mouse models. Trends Pharmacol Sci.

[50] Chan TK, Daniel Tan WS, Peh HY (2017). Aeroallergens induce reactive oxygen species production and DNA damage and dampen antioxidant responses in bronchial epithelial cells. J Immunol.

[51] Qu L, Chen C, Chen Y (2019). High-mobility group box 1 (HMGB1) and autophagy in acute lung injury (ALI): a review. Med Sci Monit.

[52] Rebetz J, Semple JW, Kapur R (2018). The pathogenic involvement of neutrophils in acute respiratory distress syndrome and transfusion-related acute lung injury. Transfus Med Hemother.

[53] Bi YW, Li LS, Ru N, Zhang B, Lei X (2024). Nicotinamide adenine dinucleotide phosphate oxidase in pancreatic diseases: Mechanisms and future perspectives. World J Gastroenterol.

[54] Mancias JD, Wang X, Gygi SP, Harper JW, Kimmelman AC (2014). Quantitative proteomics identifies NCOA4 as the cargo receptor mediating ferritinophagy. Nature.

[55] Goodall M, Thorburn A (2014). Identifying specific receptors for cargo-mediated autophagy. Cell Res.

