# Supplementary material for: Plasma exosomal miR-25-3p alleviates acute pancreatitis-associated lung injury by inhibiting ferroptosis of type II alveolar epithelial cells via NOX4/NCOA4-mediated ferritinophagy
Source: J Transl Int Med. 2026 Oct 23;14(5):799–821. doi: 10.1515/jtim-2026-0071 (PMC13726662; doi:10.1515/jtim-2026-0071)
Supplement: Supplementary file 1 [file jtim-2026-0071_sm.pdf]

Supplementary Materials

Figure S1

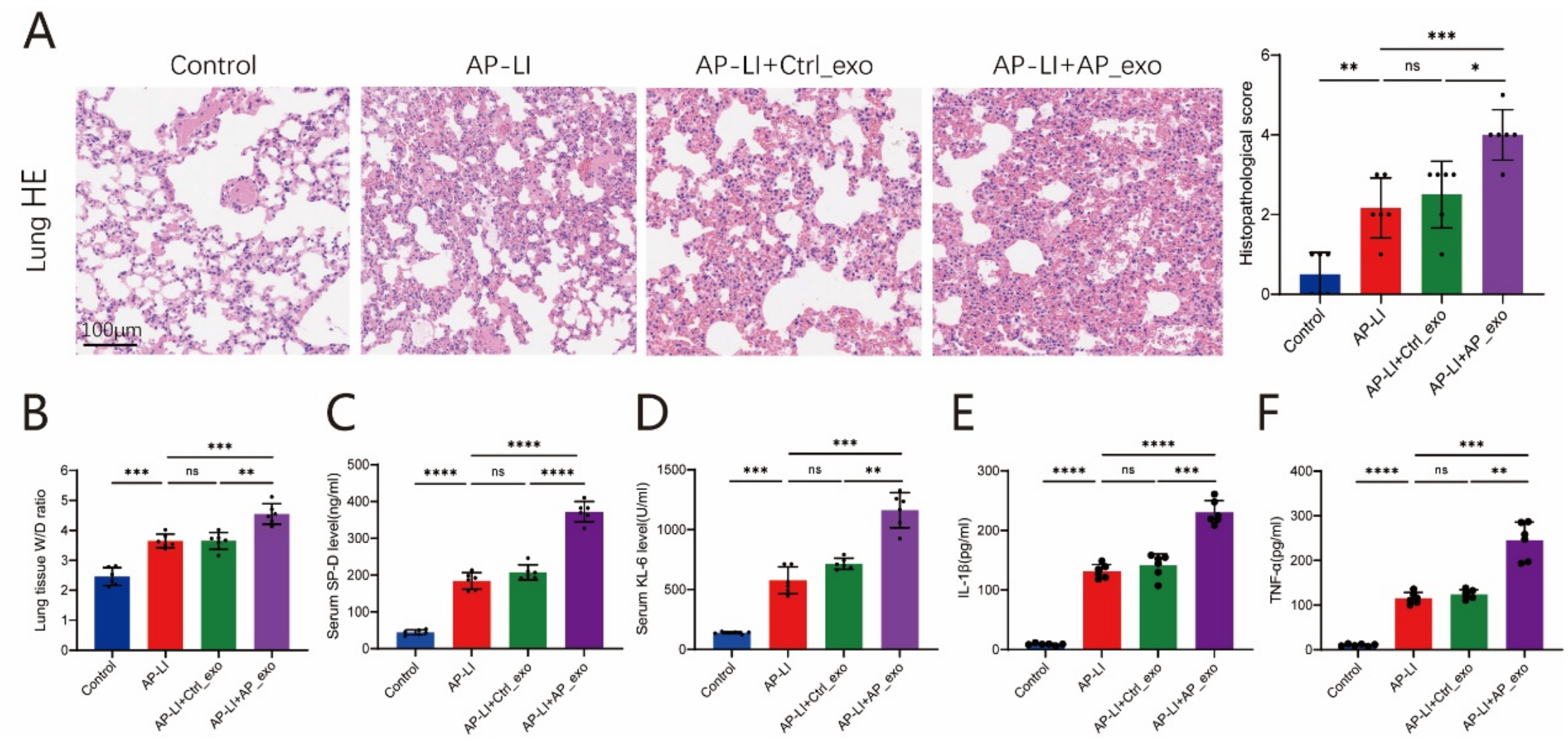

Figure S1. Plasma exosomes from AP-LI animal models aggravate AP-LI.

(A) Representative H&E staining images of lung sections from control, AP-LI, AP-LI + ctrl_exo and AP-LI + AP_exo mice. Scale bar = 100 μm. (B-F) W/D ratios and serum levels of SP-D, KL-6, IL-1β, and TNF-α in control, AP-LI, AP-LI + ctrl_exo and AP-LI + AP_exo mice. The data are presented as the mean ± SD (n=6). Statistical significance was determined by one-way ANOVA followed by Tukey's multiple comparisons test. **P*<0.05, ***P*<0.01, ****P*<0.001, *****P*<0.0001, ns not significant.

Figure S2

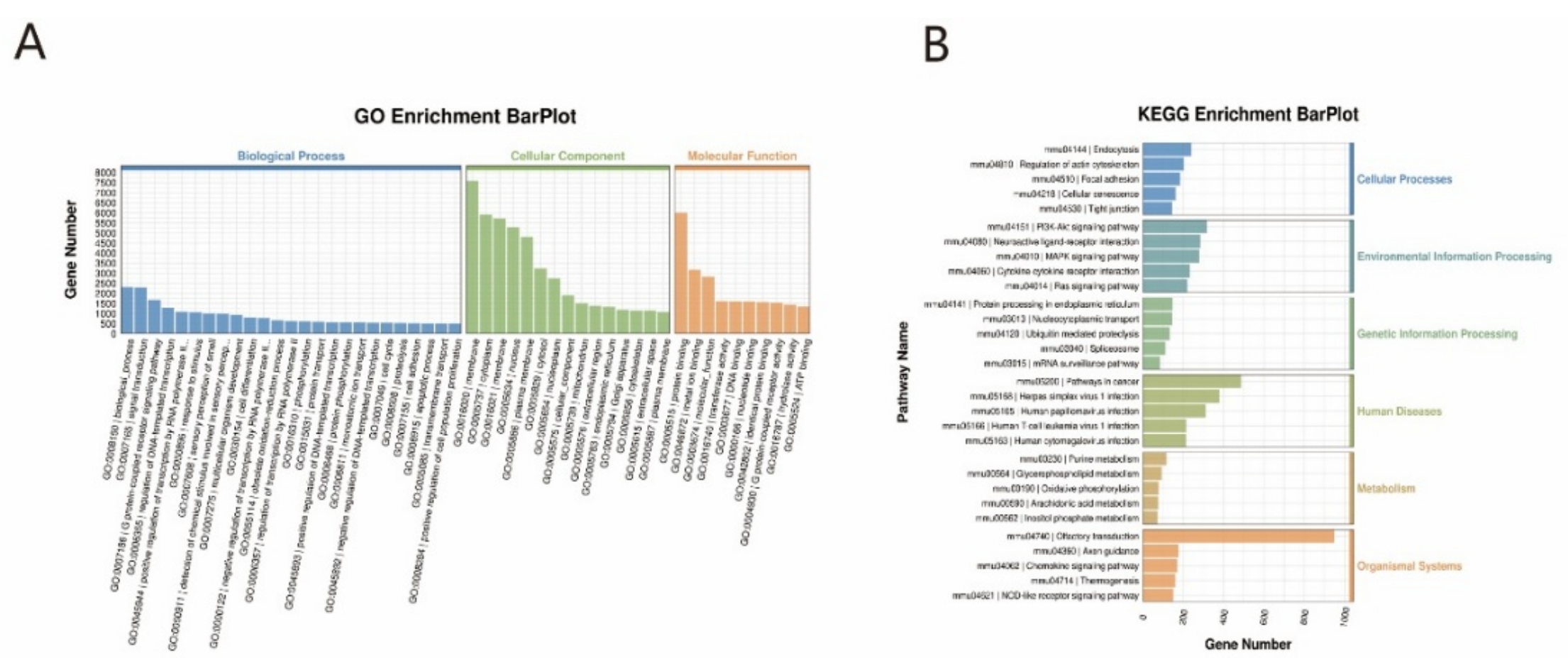

Figure S2. GO enrichment and KEGG enrichment of the miRNA sequencing results.

(A) GO enrichment bar plot of the miRNA sequencing result. **(B)** KEGG enrichment bar plot of the miRNA sequencing results.

Figure S3

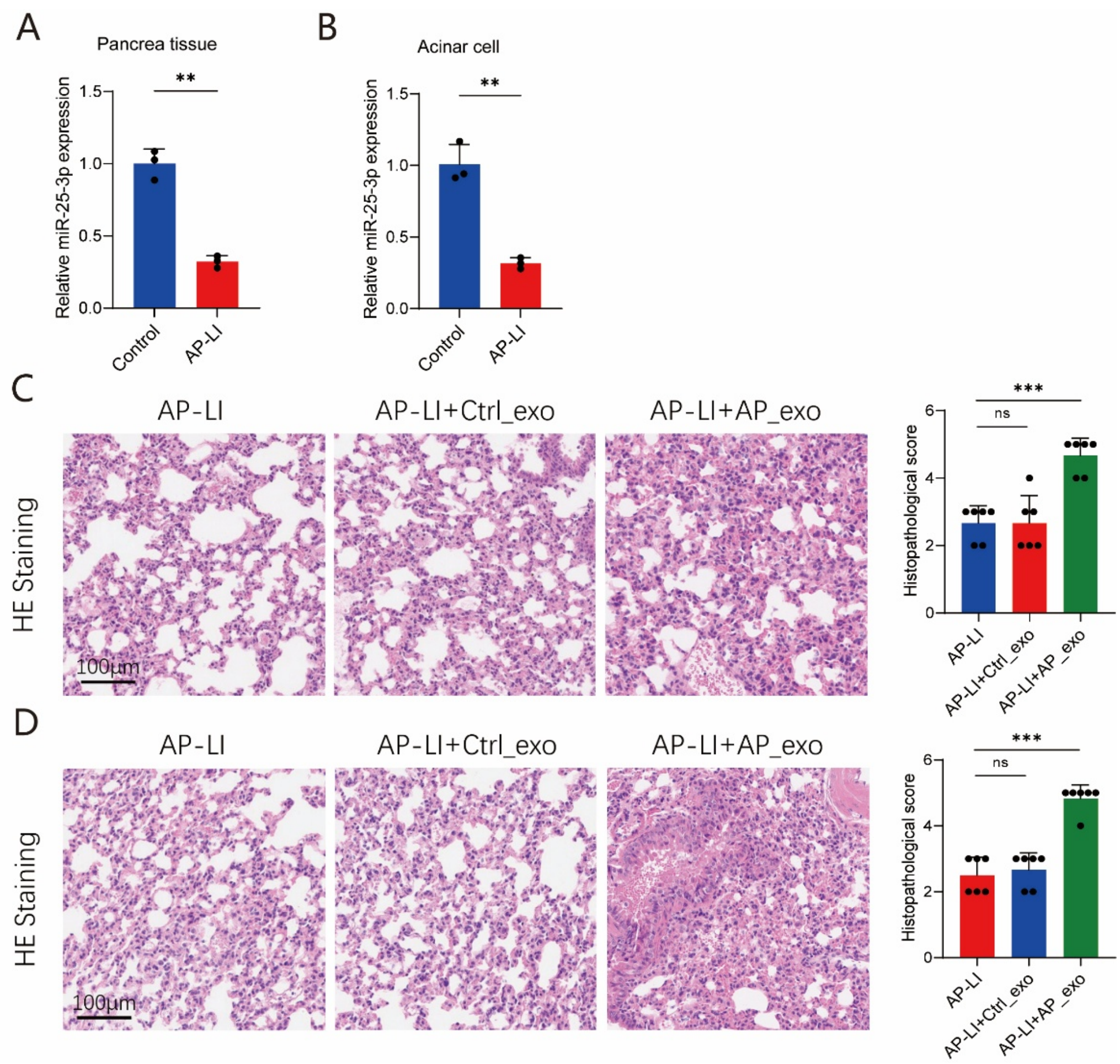

Figure S3. Exosomes isolated from pancreatic tissue and acinar cells in the AP-LI model exacerbate acute pancreatitis-associated lung injury.

(A) Exosomal miR-25-3p levels in pancreatic tissues collected from mice. **(B)** Exosomal miR-25-3p levels in primary pancreatic acinar cells isolated from mice. **(C)** Representative H&E staining images of lung tissues and histological scores from AP-LI mice injected with tissue-derived exosomes. Bar = 100 μm. **(D)** Representative H&E staining images of lung tissues and histological scores from AP-LI mice injected with primary acinar cell-derived exosomes. Bar = 100 μm. The

data are presented as the mean ± SD (A, B: n=3; C, D: n=6). Statistical significance was determined by one-way ANOVA followed by Tukey's multiple comparisons test. **P*<0.05, ***P*<0.01, ****P*<0.001, *****P*<0.0001, ns not significant.

Figure S4

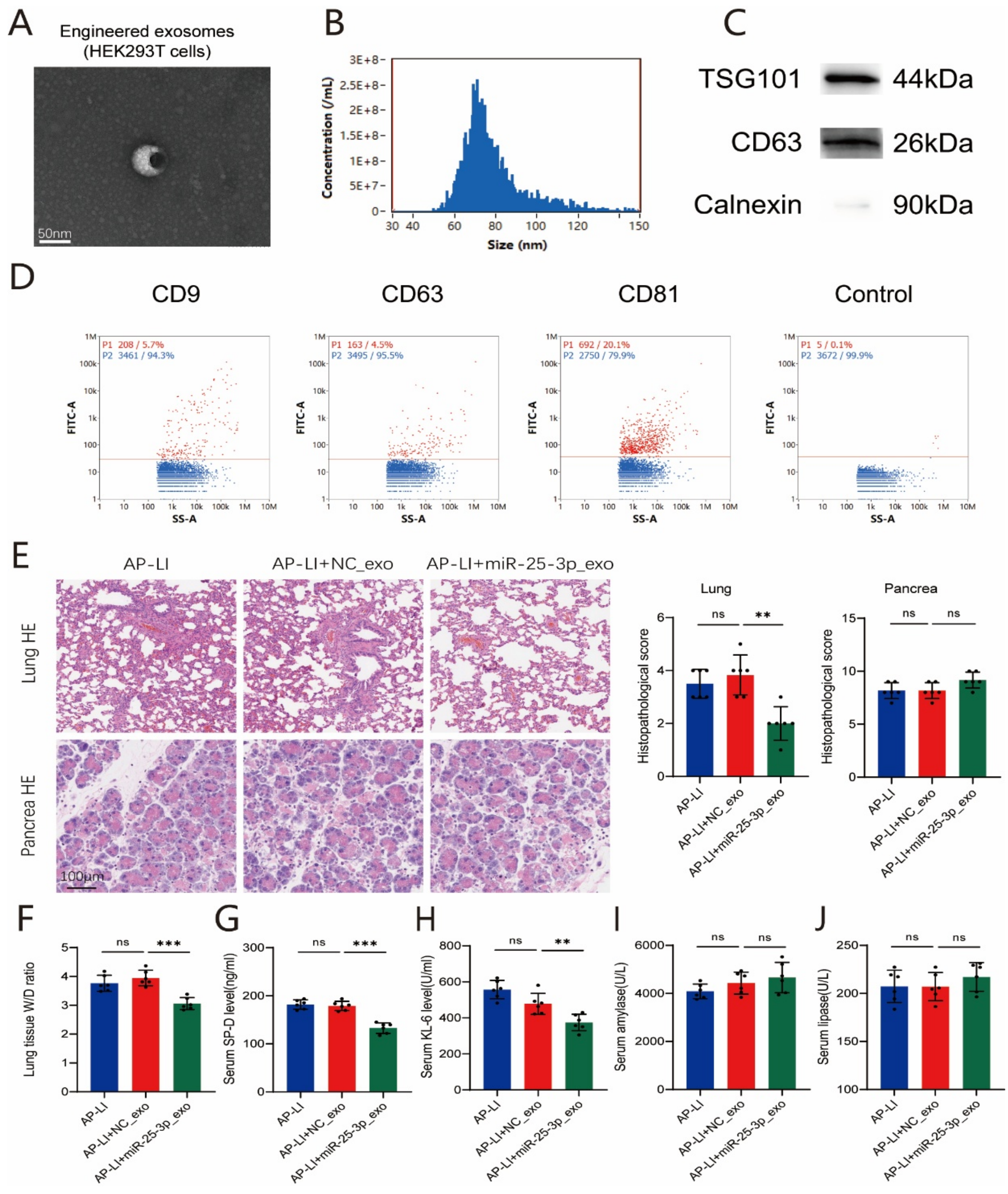

Figure S4. Administration of engineered exosomes overexpressing miR-25-3p attenuates lung tissue injury in AP-LI without significantly altering pancreatic injury.

(A) Representative TEM images of engineered exosomes from HEK293T cells. Scale bar = 50 nm.

(B) Particle size and concentration of engineered exosomes analyzed by nanoparticle tracking. **(C)** Protein expression levels of engineered exosome markers (including TSG101, CD63 and calnexin). **(D)** Surface marker analysis (CD9, CD63, and CD81) of engineered exosomes using nanoflow cytometry. **(E)** Representative H&E staining images and histological scores of lung and pancreatic tissues from AP-LI mice injected with HEK293T-derived engineered exosomes. Bar = 100 μm. **(F-H)** W/D ratios and serum levels of SP-D and KL-6 in AP-LI, AP-LI + NC_exo and miR-25-3p_exo mice. **(I, J)** Serum amylase and lipase levels in AP-LI, AP-LI + NC_exo and miR-25-3p_exo mice. The data are presented as the mean ± SD (n=6). Statistical significance was determined by one-way ANOVA followed by Tukey's multiple comparisons test. **P*<0.05, ***P*<0.01, ****P*<0.001, *****P*<0.0001, ns not significant.

Figure S5

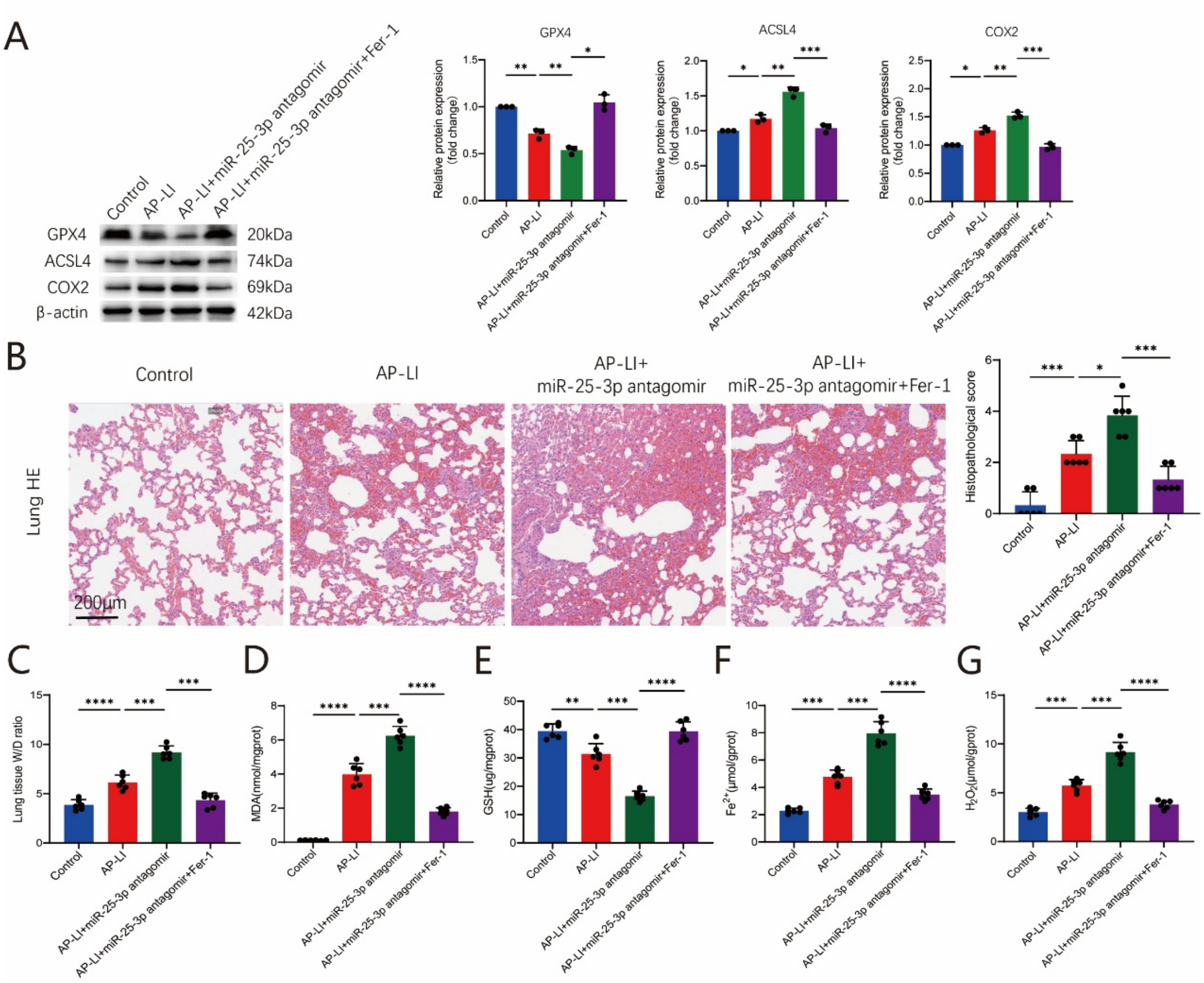

Figure S5. Plasma exosomal miR-25-3p alleviates AP-LI by inhibiting ferroptosis in vivo and in vitro.

(A) Representative Western blot images and quantitative analysis of GPX4, ACSL4, and COX2 protein expression in lung tissues from control, AP-LI, AP-LI + miR-25-3p antagomir and AP-LI + miR-25-3p antagomir + Fer-1 groups; β-actin served as a loading control. **(B)** Representative H&E staining images of lung tissues and histopathological scores for the control, AP-LI, AP-LI + miR-25-3p antagomir, and AP-LI + miR-25-3p antagomir + Fer-1 groups. **(C-G)** Lung tissue W/D ratios and MDA, GSH, Fe^{2+} and H_2O_2 levels in the control, AP-LI, AP-LI + miR-25-3p antagomir, and AP-LI + miR-25-3p antagomir + Fer-1 groups. The data are presented as the mean ± SD (A: n=3; B-G: n=6). Statistical significance was determined by one-way ANOVA followed by Tukey's multiple comparisons test. **P*<0.05, ***P*<0.01, ****P*<0.001, *****P*<0.0001, ns not significant.

Figure S6

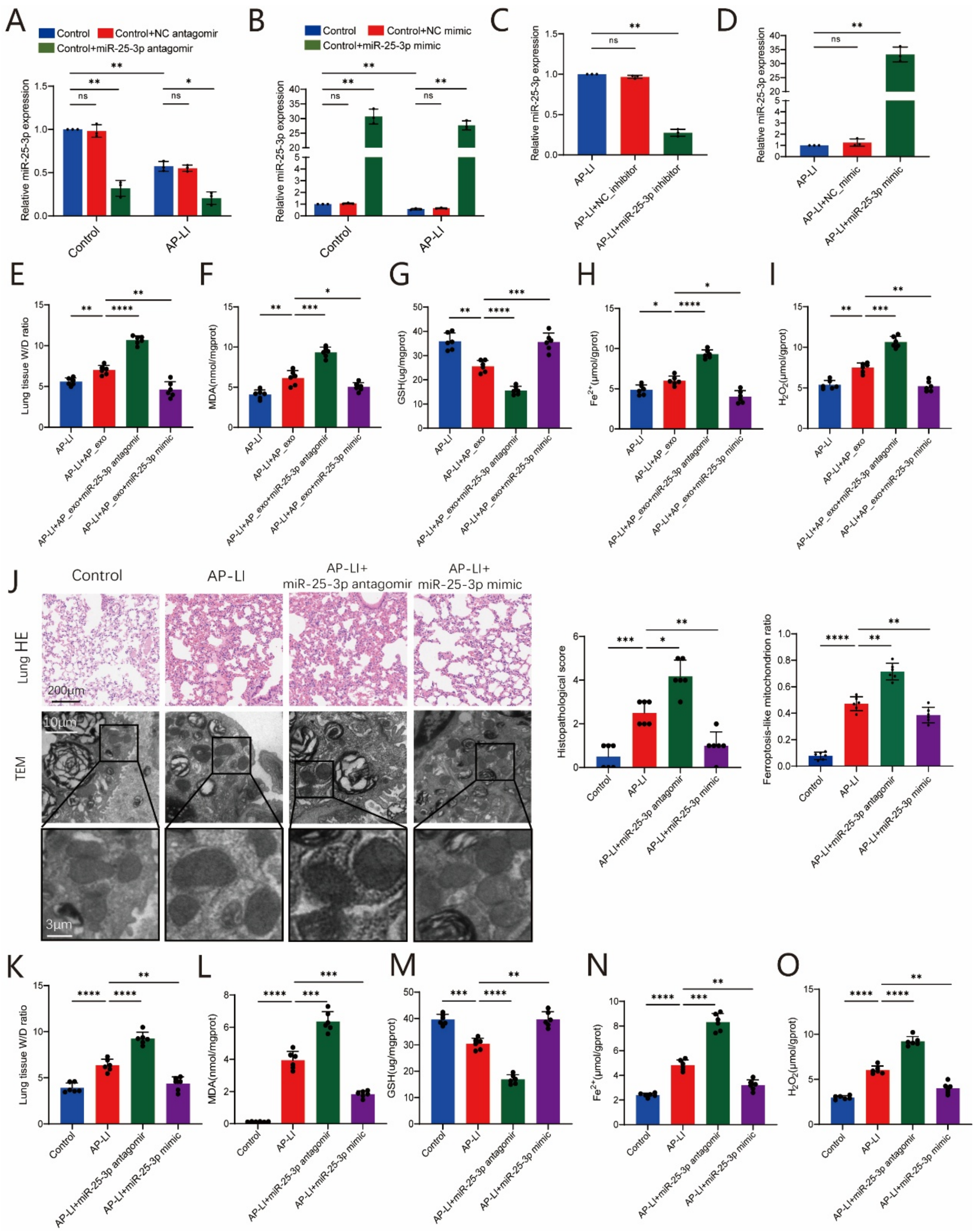

Figure S6. Plasma exosomal miR-25-3p alleviates AP-LI by inhibiting ferroptosis in AT2 cells.

(A, B) qPCR analysis of miR-25-3p expression in lung tissues from control and AP-LI mice treated with the NC antagomir, miR-25-3p antagomir, NC mimic, or miR-25-3p mimic. **(C, D)** qPCR analysis of miR-25-3p levels in AT2 cells transfected with the NC inhibitor, miR-25-3p inhibitor,

NC mimic, or miR-25-3p mimic. **(E-I)** Lung tissue W/D ratios and MDA, GSH, Fe^{2+} and H_2O_2 levels in AP-LI, AP-LI + AP_exo, AP-LI + AP_exo + miR-25-3p antagomir and AP-LI + AP_exo + miR-25-3p mimic mice. **(J)** Representative H&E staining images of lung tissues and histopathological scores in control, AP-LI, AP-LI + miR-25-3p antagomir and AP-LI + miR-25-3p mimic mice. Scale bar = 200 μm. Representative TEM images of AT2 cells. Scale bar = 10 μm. **(K-O)** Lung tissue W/D ratios and MDA, GSH, Fe^{2+} and H_2O_2 levels in control, AP-LI, AP-LI + miR-25-3p antagomir and AP-LI + miR-25-3p mimic mice. The data are presented as the mean ± SD (n=6). Statistical significance was determined by one-way ANOVA followed by Tukey's multiple comparisons test. *P<0.05, **P<0.01, ***P<0.001, ****P<0.0001, ns not significant.

Figure S7

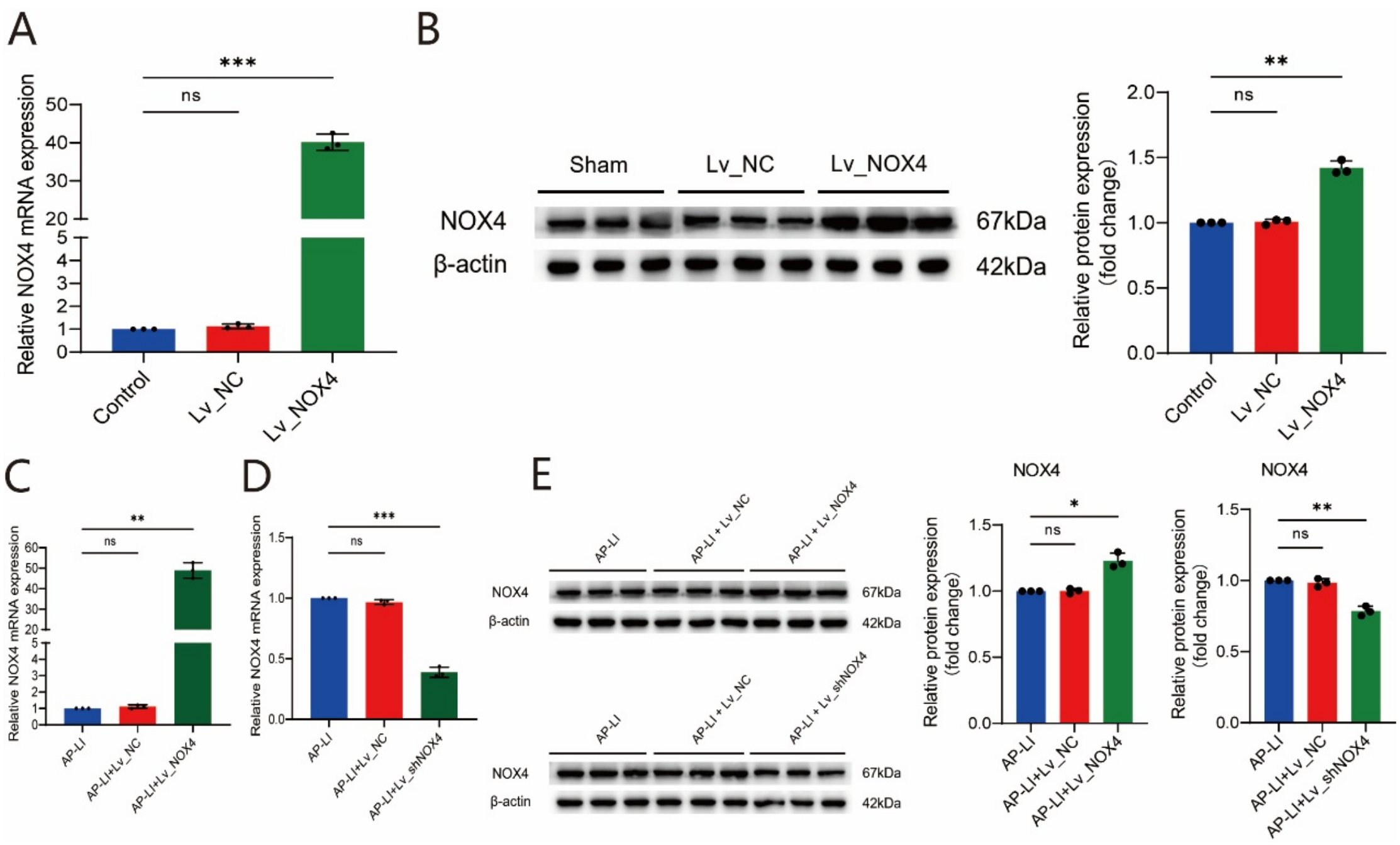

Figure S7. Overexpression and knockdown of NOX4 in vivo and in vitro.

(A, B) NOX4 mRNA and protein levels in lung tissues after lentiviral overexpression of NOX4. **(C-E)** mRNA and protein levels of NOX4 in transduced AT2 cells. β-actin was used as a loading control for Western blotting. The data are presented as the mean ± SD (n=3). Statistical significance was

determined by one-way ANOVA followed by Tukey's multiple comparisons test. *$P<0.05$, **$P<0.01$, ***$P<0.001$, ****$P<0.0001$, ns not significant.

Figure S8

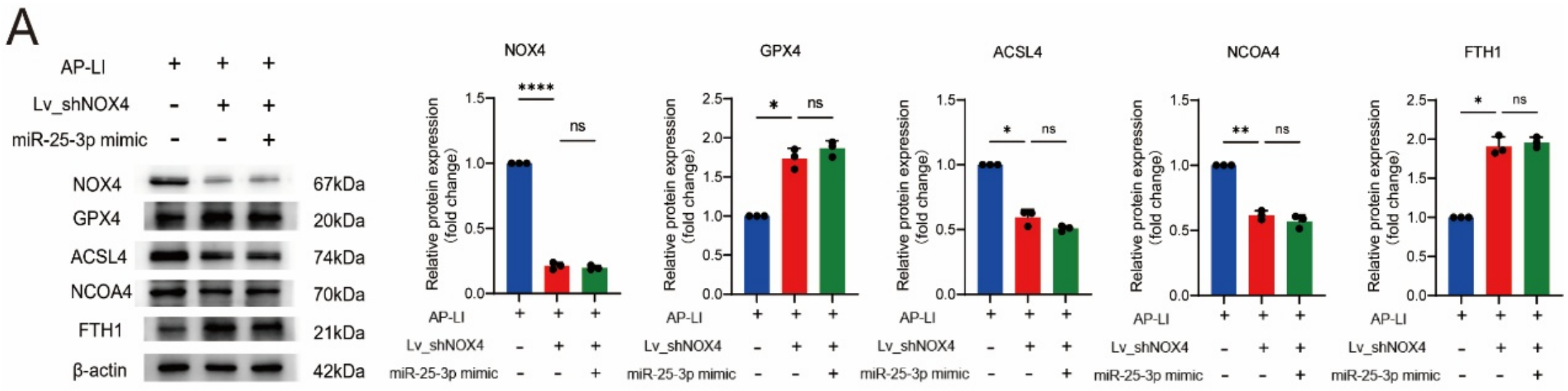

Figure S8. The miR-25-3p mimic attenuates NOX4-mediated ferroptosis in vivo and in vitro.

(A) Representative Western blot images and quantitative analysis of NOX4, GPX4, ACSL4, NCOA4 and FTH1 expression in AT2 cells that were treated with AP-LI, AP-LI + Lv_shNOX4 and AP-LI + Lv_shNOX4 + miR-25-3p mimic. β-actin was used as the protein loading control. The data are presented as the mean ± SD (n=3). Statistical significance was determined by one-way ANOVA followed by Tukey's multiple comparisons test. *$P<0.05$, **$P<0.01$, ***$P<0.001$, ****$P<0.0001$, ns not significant.

Supplementary Table 1

Table S1. shRNA target sequences.

Name	Sequences
shNOX4-1#	CTGGACCTTTGTGCCTTTATT
shNOX4-2#	GCAAGACCTGGCCAGTATATT
shNOX4-3#	ACATTTGGTGTCCACTTTAAA
shNOX4-NC	CAACAAGATGAAGAGCACCAA
shNCOA4-1#	GAAACCAGCGAGAAGTTTAAA
shNCOA4-2#	GAGAAGGAAGCCATGTATAAA
shNCOA4-3#	CACAGGAAGCATTTCTTAATT
shNCOA4-NC	CAACAAGATGAAGAGCACCAA

Supplementary Table 2

Table S2. Pathological scoring criteria for lung injury.

Score	Scoring criteria
0	Normal alveolar wall; no thickening, inflammation, or congestion.
1	Mild diffuse infiltration of neutrophils; no alveolar wall thickening.
2	Moderate infiltration with mild wall thickening (1-2 × normal).
3	Severe infiltration and wall thickening (3-5 × normal).
4	Infiltration with 25-50% lung parenchyma involvement.
5	Infiltration with >50% lung parenchyma involvement.

Supplementary Table 3

Table S3. Histopathological scoring system for pancreatic injury (Schmidt score).

Parameter	Score	Histopathological criteria
Edema	0	No edema
	1	Mild interlobular edema
	2	Moderate interlobular edema with expansion of interstitial spaces
	3	Severe, diffuse edema with marked tissue separation
Inflammatory cell infiltration	0	No inflammatory cells
	1	Mild infiltration of inflammatory cells
	2	Moderate, focal inflammatory infiltration
	3	Severe, diffuse inflammatory infiltration
Acinar cell necrosis	0	No necrosis
	1	< 5% acinar necrosis
	2	5-20% acinar necrosis
	3	> 20% acinar necrosis
Hemorrhage and fat necrosis	0	Absent
	1	Focal hemorrhage or fat necrosis
	2	Multifocal hemorrhage or fat necrosis
	3	Extensive hemorrhage or fat necrosis

Total score range: 0-12

Supplementary Table 4

Table S4. Primer sequence of genes.

Gene	Primer sequence
NOX4	F:5’-CGGGATTTGCTACTGCCTCCAT-3’
	R:5’-GTGACTCCTCAAATGGGCTTCC-3’
NCOA4	F:5’-TGCCATTGGTCTTCAGGCTCCT-3’
	R:5’-CAGGCATCGCTGAAGAAACTGC-3’
FTH1	F:5’-GCCGAGAAACTGATGAAGCTGC-3’
	R:5’-GCACACTCCATTGCATTCAGCC-3’
HIF-1α	F: 5’-CAGCTGCGTGAACTCAACAT-3’
	R: 5’-TCACACGGATCCTCTTACCC-3’
GAPDH	F:5’-CATCACTGCCACCCAGAAGACTG-3’
	R:5’-ATGCCAGTGAGCTTCCCGTTCAG-3’
miR-25-3p	F:5’-ACACTCCAGCTGGGCATTGCACTTGTCTCG-3’
	R:5’-CTCAACTGGTGTCGTGGAGTCGGCAATTCAGTTGAGTCAGACCG-3’
U6	F:5’-CTCGCTTCGGCAGCACA-3’
	R:5’-AACGCTTCACGAATTTGCGT-3’

Primer sequence of NOX4, NCOA4, FTH1, HIF-1α, GAPDH, miR-25-3p and U6.

F forward primer sequence, *R* reverse primer sequence.

Supplementary Table 5

Table S5. Patient baseline characteristics.

Characteristics	Healthy controls	MAP/MSAP	SAP	*P*
n	15	15	15	
Gender				0.836
Female	8 (53.33%)	7 (46.67%)	9 (60%)	
Male	7 (46.67%)	8 (53.33%)	6 (40%)	
Age (years)	38.33 (28, 55)	42.47 (31, 57)	43.53 (30, 56)	0.478
BMI (kg/m²)	21.43 (19.35, 23.46)	23.57 (20.94, 25.53)	24.11 (21.23, 25.39)	0.066
**Underlying disease**				
Biliary disease	0 (0%)	6 (40%)	4 (26.67%)	0.019
Diabetes mellitus	2 (13.33%)	3 (20%)	4 (26.67%)	0.623
Hyperlipidemia	2 (13.33%)	8 (53.33%)	10 (66.67%)	0.005
Hypertension	4 (26.67%)	5 (33.33%)	6 (40%)	0.836
Smoking history	4 (26.67%)	4 (26.67%)	5 (33.33%)	0.918
Alcohol consumption history	5 (33.33%)	4 (26.67%)	5 (33.33%)	0.918
**Aetiology**				
Biliary	-	6 (40%)	4 (26.67%)	0.474
Hypertriglyceridemia	-	8 (53.33%)	10 (66.67%)	0.474
Others	-	1 (6.67%)	1 (6.67%)	1.000
**Hospital course/outcomes**				
ICU admission	-	1 (6.67%)	12 (80%)	<0.001
Length of hospital stay (days)	-	13.67 (8, 25)	24.47 (18, 45)	<0.001

Values are expressed as median (IQR) for continuous variables and number (percentage) for categorical variables. Statistical significance was defined as a two-tailed *P* value < 0.05. MAP, mild acute pancreatitis; MSAP, moderately severe acute pancreatitis; SAP, severe acute pancreatitis; BMI, body mass index; ICU, intensive care unit.
